# Extracellular ATP promotes breast cancer chemoresistance via HIF-1α signaling

**DOI:** 10.1038/s41419-022-04647-6

**Published:** 2022-03-02

**Authors:** Hui Yang, Yue-Hang Geng, Peng Wang, Hong-Quan Zhang, Wei-Gang Fang, Xin-Xia Tian

**Affiliations:** 1 Department of Pathology, Key Laboratory of Carcinogenesis and Translational Research (Ministry of Education), School of Basic Medical Sciences, Third Hospital, Peking University Health Science Center, Beijing, 100191 China; 2 Translational Cancer Research Center, Peking University First Hospital, Beijing, 100034 China; 3 Office of Scientific Research, Peking University Health Science Center, Beijing, 100191 China; 4 Department of Anatomy, Histology and Embryology, Peking University Health Science Center, Beijing, 100191 China

**Keywords:** Breast cancer, Cell signalling

## Abstract

We have previously demonstrated that extracellular adenosine 5'-triphosphate (ATP) promotes breast cancer cell chemoresistance. However, the underlying mechanism remains unclear. Using a cDNA microarray, we demonstrated that extracellular ATP can stimulate hypoxia-inducible factor (HIF) signaling. In this study, we report that hypoxia-inducible factor 1α (HIF-1α) was upregulated after ATP treatment and mediated the ATP-driven chemoresistance process. We aimed to investigate the mechanisms and identify potential clinically relevant targets that are involved. Using mass spectrometry, we found that aldolase A (ALDOA) interacts with HIF-1α and increases HIF-1α expression. We then demonstrated that STAT3-ALDOA mediates ATP-HIF-1α signaling and upregulates the HIF-1 target genes adrenomedullin (*ADM*) and phosphoinositide-dependent kinase-1 (*PDK1*). Moreover, we show that PI3K/AKT acts upstream of HIF-1α in ATP signaling and contributes to chemoresistance in breast cancer cells. In addition, HIF-1α-knockdown or treatment with direct HIF inhibitors combined with the ATP hydrolase apyrase in MDA-MB-231 cells induced enhanced drug sensitivity in nude BALB/c mice. We then used in vitro spheroid formation assays to demonstrate the significance of ATP-HIF-1α in mediating chemoresistance. Furthermore, considering that indirect HIF inhibitors are effective in clinical cancer therapy, we treated tumor-bearing BALB/c mice with STAT3 and PI3K/AKT inhibitors and found that the dual-targeting strategy sensitized breast cancer to cisplatin. Finally, using breast cancer tissue microarrays, we found that ATP-HIF-1α signaling is associated with cancer progression, poor prognosis, and resistance to chemotherapy. Taken together, we suggest that HIF-1α signaling is vital in ATP-driven chemoresistance and may serve as a potential target for breast cancer therapies.

## Introduction

Extracellular adenosine 5'-triphosphate (ATP) is considered to be an important messenger in mediating cell survival, proliferation, and migration, and it can act as a chemotactic molecule for the recruitment of immune phagocytes [1]. Under physiological conditions, ATP is stored in very high amounts intracellularly (5–10 mmol/L) while being present in minute amounts (nmol/L) in the extracellular space [2]. ATP can be released from cells during particular physiological and pathological processes such as neurotransmission, hypotonic stress, cell injury from inflammation, and tumor necrosis [3, 4]. Specifically, a large increase in the concentration of extracellular ATP in the tumor microenvironment (TME) has been identified as a malignant tumor phenotype [2]. Evidence suggests that extracellular ATP can regulate tumor growth and shape the TME directly by acting through receptors, mainly through P2 purinergic receptors on both tumor and host cells [5, 6]. The pro-invasive role of extracellular ATP and its underlying mechanism has been well investigated by our group and others. Over the past several decades, we demonstrated that extracellular ATP could promote cancer cell invasion via the P2Y2 and P2X7 receptors to regulate epithelial-mesenchymal transition (EMT) and invasion-associated molecules, including IL-8, E-cadherin, Snail, Claudin-1, β-catenin, S100A4, HIF-2α, and SOX9, as well as through activation of epidermal growth factor receptor (EGFR) and extracellular signal-regulated kinase 1/2 (ERK1/2) signaling [7–14].

Cancer is the leading cause of death among non-communicable diseases worldwide [15]. One of the key causes of recurrence and death is the common development of chemoresistance during cancer therapy [16]. Therefore, it is essential to find new ways to overcome this problem in clinical medicine. Recently, we found that extracellular ATP plays a vital role in mediating breast cancer chemoresistance [14]. We demonstrated that extracellular ATP could upregulate the expression of *SOX9* in breast cancer cells, inducing the expression of *SOX9* target genes to reduce drug sensitivity. However, ATP can stimulate multiple downstream effectors, and the mechanism involved in ATP-driven chemoresistance still needs to be explored further. We previously found that extracellular ATP could stimulate hypoxia-inducible factor (HIF) signaling under normoxic conditions from a cDNA microarray analysis [13] and demonstrated the upregulation of HIF-1α by extracellular ATP. HIF-1α is closely associated with resistance to drugs, including cisplatin and trastuzumab, in several carcinomas [17, 18]. Therefore, targeting HIF-1α-associated signaling could serve as a potential therapeutic target to improve the effectiveness of clinical cancer therapies. However, whether HIF-1α is the sole critical factor that ATP affects to promote chemoresistance in breast cancer cells and the underlying mechanism involved remain unclear.

HIFs are heterodimers of HIF-α (isoforms HIF-1α, HIF-2α, HIF-3α) and HIF-β (also known as aryl hydrocarbon receptor nuclear translocator [ARNT]) subunits that belong to the Per-ARNT-Sim (PAS) family of basic helix–loop–helix (bHLH) transcription factors [19]. The level of HIF-1α expression is determined by the rates of protein synthesis and degradation, as is typical for proteins [20]. Synthesis of HIF-1α is regulated via O_2_-independent mechanisms, such as growth-factor stimulation, whereas degradation is regulated primarily via O_2_-dependent mechanisms [20, 21]. A lot of work has been done to elucidate the regulation of HIF-1α under hypoxic conditions [22]; however, the regulation of O_2_-independent mechanisms in breast carcinoma remains relatively poorly investigated.

Therefore, in the current study, we investigated the role of HIF-1α in ATP-driven chemoresistance and clarified the molecular mechanisms to identify potential clinical targets involved.

## Results

### HIF-1α mediates ATP-driven chemoresistance in vitro

We have demonstrated that extracellular ATP can regulate HIF signaling by elevating HIF-1/2α expression in a time- and dose-dependent manner [13]. However, the function of the ATP-HIF-1α signaling pathway remains unclear. Evidence suggests that ATP [14] and HIF-1α are closely associated with breast cancer chemoresistance [23] as well as drug resistance in multiple other carcinomas [24, 25]. Therefore, we hypothesized that HIF-1α may play an important role in ATP-driven chemoresistance.

**Fig. 1:**
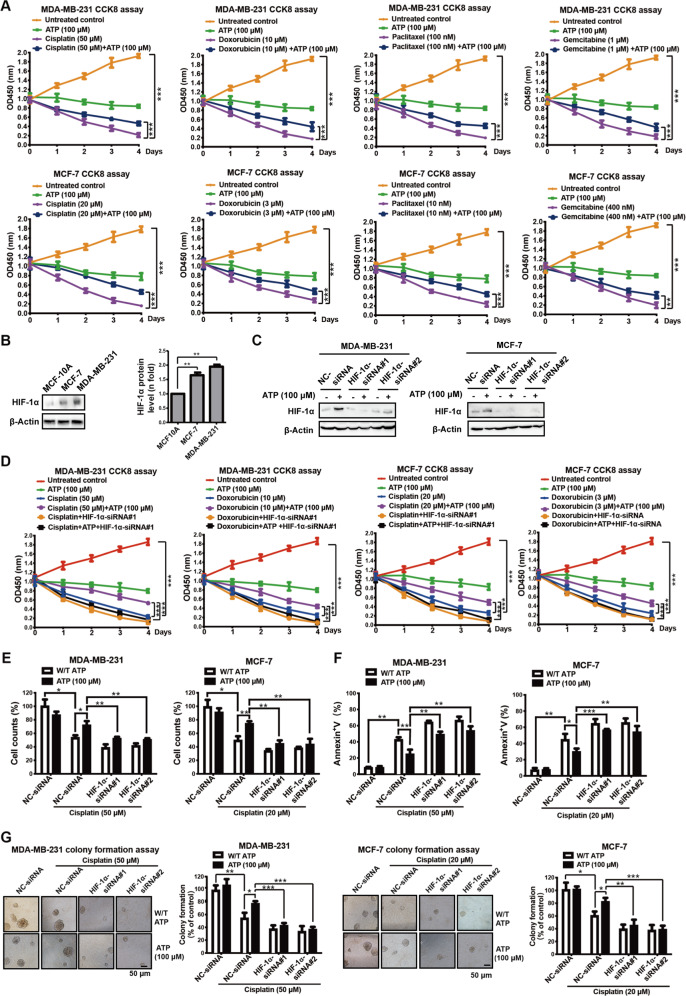
Extracellular ATP promotes breast cancer chemoresistance via HIF-1α. **A** CCK-8 assay measured by absorbance OD450 illustrated that ATP enhanced the chemotherapy resistance of cisplatin, doxorubicin, paclitaxel, and gemcitabine in MDA-MB-231 (upper) and MCF-7 cells (lower). **B** Western blotting showed expressions of HIF-1α in MCF-10A, MCF-7, and MDA-MB-231 cell lines. **C** Western blotting demonstrated expression of HIF-1α was knocked down in MDA-MB-231 and MCF-7 cells. **D** MDA-MB-231 and MCF-7 cells were transfected with NC-siRNA or HIF-1α-siRNA for 48 h, followed by cisplatin/doxorubicin treatment alone or in combination with ATP for a further 4 days. CCK-8 assay proved that ATP-promoted cisplatin/doxorubicin resistance was attenuated by HIF-1α-siRNA. **E, F** MDA-MB-231 and MCF-7 cells were transfected with NC-siRNA or HIF-1α-siRNA for 48 h, followed by cisplatin treatment alone or in combination with ATP for a further 2 days. Cell counting via trypan blue dye exclusion assay (**E**) and annexin V/PI dual staining assays (**F**) proved that ATP-promoted cisplatin resistance was attenuated by HIF-1α-siRNA. **G** MDA-MB-231 and MCF-7 cells were transfected with NC-siRNA or HIF-1α-siRNA or treated with cisplatin or ATP twice a week. Clonogenic assay (3 weeks) in 6-well plate showed that HIF-1α-siRNA attenuated ATP-enhanced cisplatin resistance of MDA-MB-231 and MCF-7 cells. Data are representative of at least three independent experiments. Error bars represent means ± SD from triplicate experiments. *p < 0.05; **p < 0.01; ***p < 0.001; ns, not significant.

To further investigate the generalized effects of ATP-driven chemoresistance in breast cancer, we performed a CCK-8 assay in triple-negative breast cancer MDA-MB-231 cells and estrogen receptor (ER)-positive breast cancer MCF-7 cells treated with standard chemotherapy drugs for breast carcinomas, such as cisplatin, doxorubicin, paclitaxel, and gemcitabine [26] at the concentration of each drugs’ IC_50_ at 48 h, with or without 100 μM extracellular ATP, a concentration that induced significant chemoresistance in our previous study [14]. We found that ATP increased cell survival under the various drug treatments (Fig. 1A).

To further investigate the possible role of ATP-HIF-1α signaling in breast cancer chemoresistance, we detected the expression of HIF-1α in MDA-MB-231, MCF-7, and MCF-10A (normal breast epithelial cells) (Fig. 1B). HIF-1α expression was higher in MDA-MB-231 and MCF-7 cells than in MCF-10A cells. Therefore, we knocked down HIF-1α in MDA-MB-231 and MCF-7 cells using two independent HIF-1α-siRNAs (Fig. 1C) and found attenuated ATP-induced cisplatin and doxorubicin resistance (Fig. 1D). We chose to continue the study using cisplatin, a vital drug for treating triple-negative breast carcinoma [27], metastatic breast carcinoma [13], and other forms of carcinomas [17, 28] for further study. We next demonstrated the vital role of ATP-HIF-1α in mediating cisplatin resistance by trypan blue dye exclusion and cell counting assays (Fig. 1E) as well as assessment of apoptosis by annexin V/propidium iodide (PI) dual staining (Fig. 1F). To further explore the role of ATP-HIF-1α in long-term drug treatment, we introduced HIF-1α-siRNAs into cells and then conducted a colony-formation assay with or without cisplatin treatment for 3 weeks. Knockdown of HIF-1α was sufficient to suppress ATP-driven cisplatin-resistant colony formation in MDA-MB-231 and MCF-7 cells (Fig. 1G). These results indicate that HIF-1α mediates ATP-driven chemoresistance in vitro.

### HIF-1α interacts with ALDOA and is regulated by ATP-STAT3-ALDOA

**Fig. 2:**
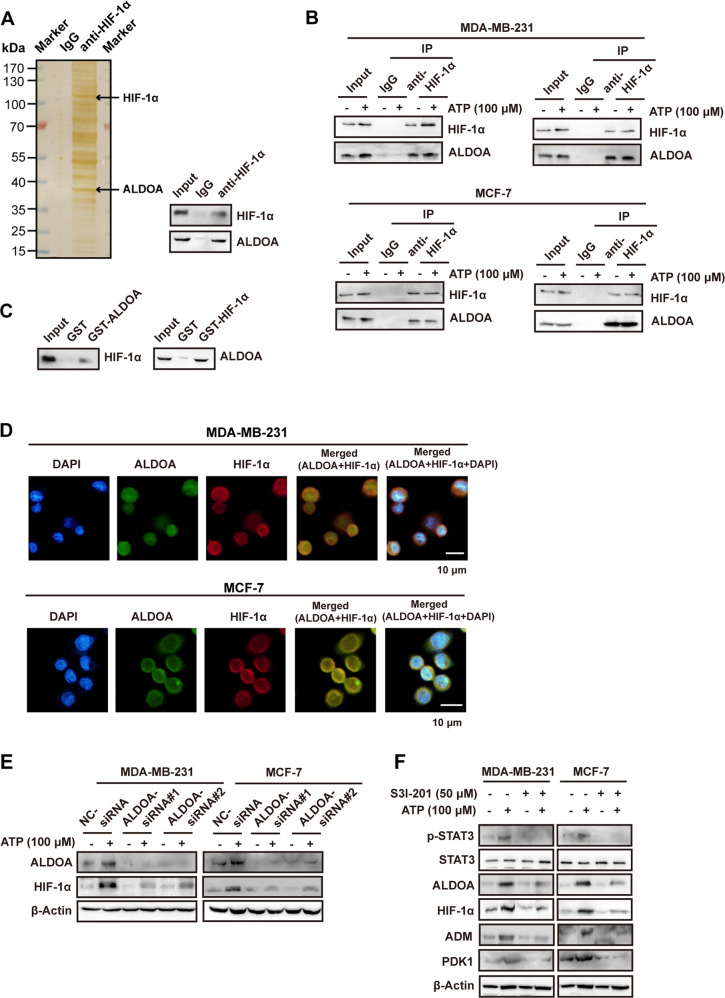
ALDOA interacts with HIF-1α and STAT3-ALDOA mediates ATP-HIF-1α signaling. **A** MCF-7 cells lysed and co-immunoprecipitated (co-IP) with anti-immunoglobulin G (IgG) or anti-HIF-1α. elutes were resolved by SDS-PAGE and silver-stained (left). The presence of ALDOA was detected by mass spectrometry and demonstrated by western blotting (right). **B** Physical interaction between ALDOA and HIF-1α was determined by co-immunoprecipitation and western blotting in both MDA-MB-231 and MCF-7 cells. **C** The interaction between ALDOA and HIF-1α was demonstrated by GST pull-down assays with bacterially expressed GST-fused ALDOA and endogenous transcribed/translated HIF-1α (left), or with GST-fused HIF-1α and endogenous transcribed/translated ALDOA (right). **D** MDA-MB-231 and MCF-7 cells were double-stained with ALDOA (green) and HIF-1α (red), counterstained with DAPI (blue), and observed under a confocal microscope. The merged regions indicated their co-localization. **E** Western blotting proved that the elevation of HIF-1α via ATP was attenuated by ALDOA-siRNAs in MDA-MB-231 and MCF-7 cells. **F** Western blotting illustrated that the expression changes of ATP-HIF-1α signaling were attenuated by S3I-201 (STAT3 inhibitor) in MDA-MB-231 and MCF-7 cells. Data are representative of at least three independent experiments. Error bars represent means ± SD from triplicate experiments. *p < 0.05; **p < 0.01; ***p < 0.001; ns, not significant.

To further investigate the mechanism involved in HIF-1α signaling, we performed mass spectrometry to detect proteins interacting with HIF-1α. Cellular extracts from MCF-7 cells were co-immunoprecipitated (co-IP) with immunoglobulin G (IgG) or anti-HIF-1α antibody, resolved by sodium dodecyl sulfate–polyacrylamide gel electrophoresis (SDS-PAGE), and visualized using silver staining (Fig. 2A). Protein bands on the gel were recovered and analyzed by mass spectrometry. One identified protein was aldolase A (ALDOA), which is associated with HIF signaling and poor prognosis in human carcinomas [29, 30]. The presence of ALDOA in the interactome (Supplementary Table 1) was verified by western blotting of the eluate (Fig. 2A, right).

To confirm this result, we demonstrated the interaction between HIF-1α and ALDOA with or without ATP treatment by co-IP in MCF-7 and MDA-MB-231 cells (Fig. 2B). Glutathione S-transferase (GST) pull-down assays verified the direct interaction between HIF-1α and ALDOA (Fig. 2C). Moreover, we observed co-localization of HIF-1α and ALDOA in the cell via immunofluorescence (Fig. 2D), which further suggests their interaction.

To determine the potential role of ALDOA in HIF-1α regulation after ATP treatment, we knocked down ALDOA with two independent ALDOA-siRNAs. We found that ATP could upregulate ALDOA, and the knockdown of ALDOA reduced the ATP-driven upregulation of HIF-1α in MDA-MB-231 and MCF-7 cells (Fig. 2E), indicating the involvement of ALDOA in mediating ATP-HIF-1α signaling.

Evidence suggests that ALDOA is a candidate marker for STAT3-targeting therapy [31]. To explore the regulatory mechanism of ALDOA in ATP-induced signaling, we used the STAT3 inhibitor S3I-201 to block phosphorylation of STAT3 (Tyr 705) and found that it attenuated the ATP-driven upregulations of ALDOA and HIF-1α, as well as the HIF-1 target genes adrenomedullin (*ADM*) [32] and phosphoinositide-dependent kinase-1 (*PDK1*) [33] (Fig. 2F). *ADM* and *PDK1* were also upregulated in a cDNA microarray of ATP-treated cells [13]. These results suggest that STAT3 regulates the ATP-HIF-1α signaling.

### PI3K/AKT axis and P2Y2 receptor regulate ATP-HIF-1α signaling

**Fig. 3:**
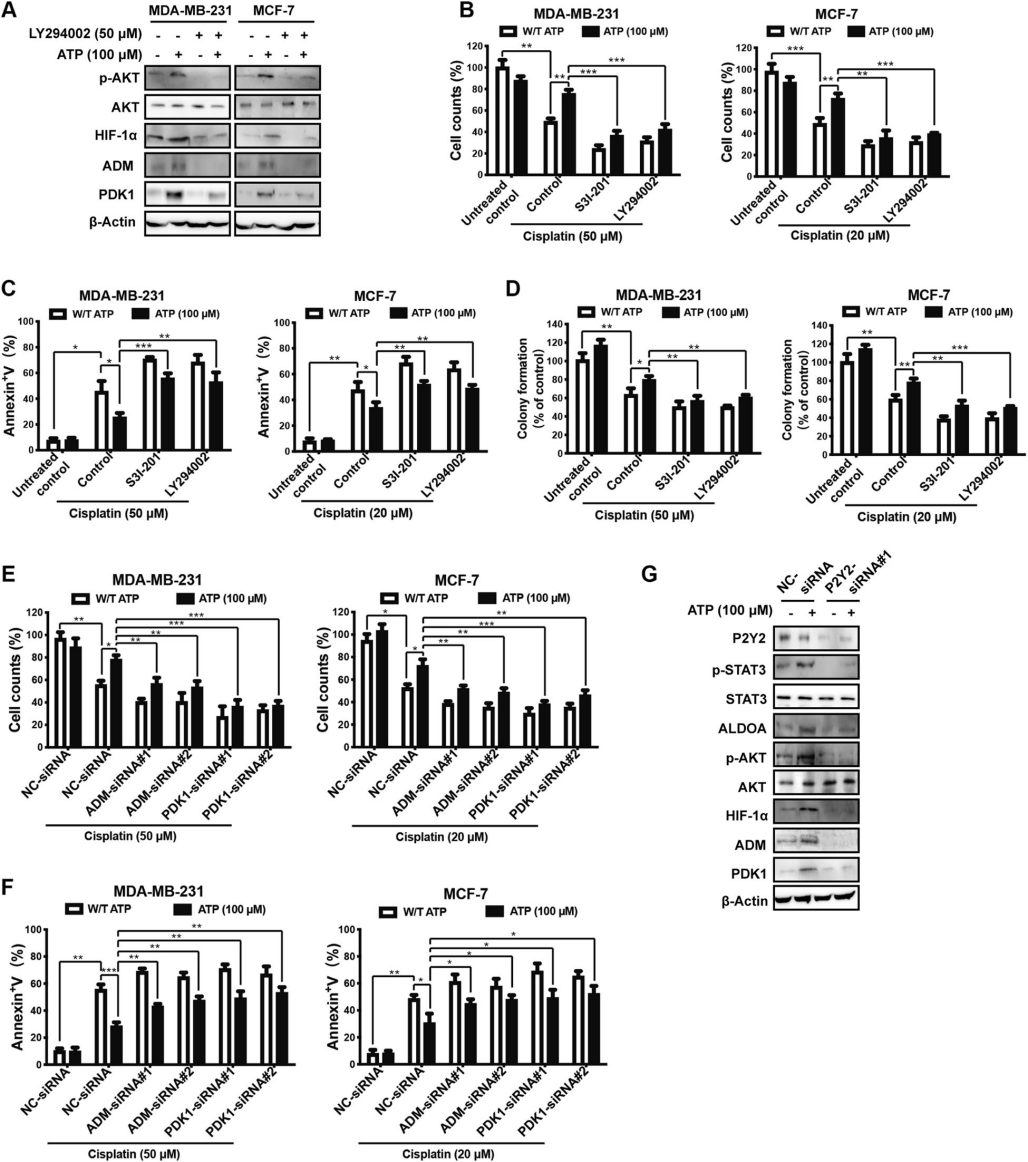
ATP regulates HIF-1α signaling *via* PI3K/AKT pathway and P2Y2 receptor. **A** Western blotting illustrated that ATP-driven HIF-1α and HIF-1α target proteins were attenuated by LY294002 (PI3K/AKT inhibitor). **B–D** MDA-MB-231 and MCF-7 cells were treated with/without cisplatin plus S3I-201 or LY294002 for 2 days (**B**, **C**) or 3 weeks (**D**). Cell counting via trypan blue dye exclusion assay (**B**), Annexin V/PI dual staining assay (**C**), and clonogenic assay (**D**) showed that ATP-driven cisplatin resistance was attenuated by S3I-201 or LY294002. **E, F** MDA-MB-231 and MCF-7 cells were transfected with NC-siRNA or ADM-siRNA or PDK1-siRNA for 48 h, followed by cisplatin treatment alone or in combination with ATP for a further 2 days. Cell counting via trypan blue dye exclusion assay (**E**) and Annexin V/PI dual staining assay (**F**) showed that ATP-driven cisplatin resistance was attenuated by ADM-siRNA or PDK1-siRNA. **G** Western blotting proved that P2Y2-siRNA attenuated ATP-driven expression alterations in HIF-1α signaling. Data are representative of at least three independent experiments. Error bars represent means ± SD from triplicate experiments. *p < 0.05; **p < 0.01; ***p < 0.001; ns, not significant.

We previously demonstrated that HIF-1α is regulated by AKT signaling [34]. In addition, we demonstrated that extracellular ATP stimulates phosphatidylinositol 3-kinase (PI3K)/AKT signaling [9]. Therefore, we hypothesized that PI3K/AKT signaling was involved in this process and tested the dose-dependent effect of the PI3K inhibitor LY294002 on PI3K/AKT signaling [35]. Treatment with 50 μM LY294002 resulted in maximal inhibition of AKT (T308) and HIF-1α (Fig. S1A). We separately used 50 μM LY294002 (Fig. 3A) and AKT-siRNAs (Fig. S1B) and found that both inhibited expressions of the HIF-1α and HIF-1 target genes *ADM* and *PDK1*, suggesting the involvement of HIF-1α signaling in the ATP-PI3K/AKT pathway.

To determine the function of both STAT3 and AKT signaling upstream of HIF-1α as well as the HIF-1α target genes *ADM* and *PDK1* in ATP-induced drug resistance, we performed cell counting (Fig. 3B), apoptosis (Fig. 3C), and colony formation assays (Fig. 3D) using S3I-201 and LY294002 along with cisplatin treatment. In addition, we introduced siRNAs against ADM and PDK1 and then performed cell counting (Fig. 3E) and apoptosis assays (Fig. 3F) after cisplatin treatment. We found that inhibiting STAT3 or AKT signaling or knocking down either of the HIF-1α target genes *ADM* or *PDK1* could attenuate ATP-driven survival and apoptosis resistance under cisplatin treatment (Fig. 3B–F). These findings build on our previous work where we demonstrated the vital role of STAT3 in breast cancer chemoresistance [14]. Altogether, evidence suggests that ATP-HIF-1α signaling contributes to ATP-driven breast cancer chemoresistance.

We previously demonstrated the important role of the P2Y2 receptor in mediating ATP-related biological processes [7, 10, 14]. Therefore, we next investigated whether the P2Y2 receptor is involved in ATP-HIF-1α signaling. After knocking down P2Y2 using P2Y2-siRNA, the ATP-induced phosphorylation levels of STAT3 and AKT as well as upregulation of HIF-1α and its target genes were attenuated (Fig. 3G). Taken together, our data suggest that the PI3K/AKT and P2Y2 receptors are involved in regulating ATP-HIF-1α signaling.

### ATP-HIF-1α signaling promotes xenograft tumor chemoresistance in vivo

**Fig. 4:**
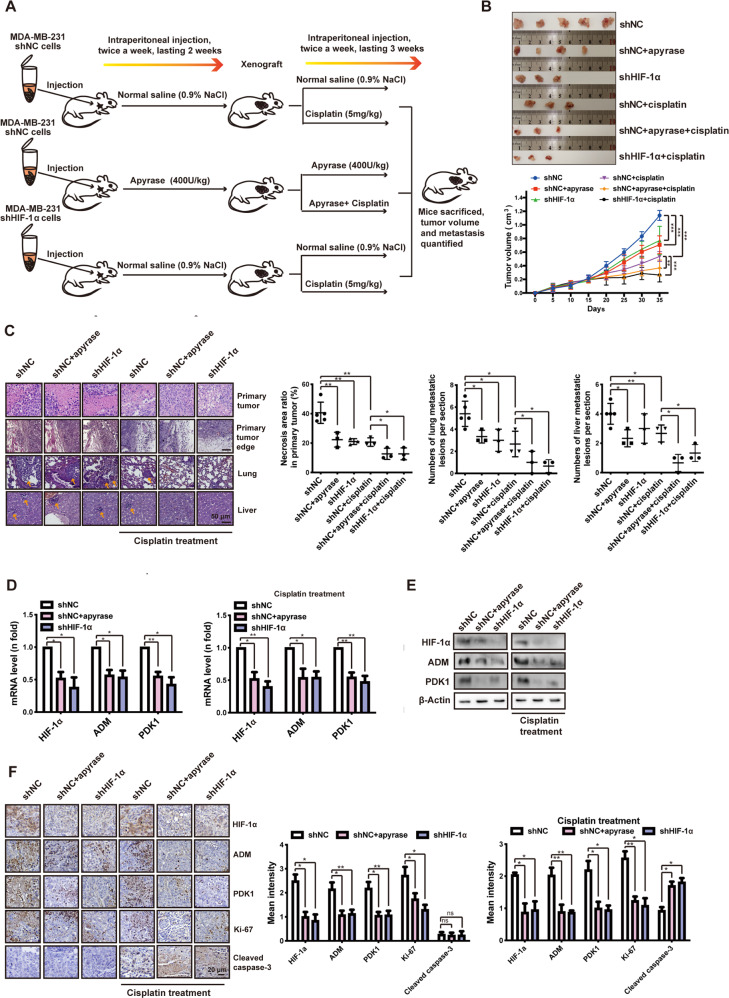
ATP-HIF-1α signaling promotes xenograft tumor chemoresistance in vivo. **A** A model of xenograft experiment. In brief, six million stably transfected MDA-MB-231 cells (shNC, shHIF-1α) were inoculated orthotopically onto the mammary fat pad of 6-week-old female Balb/c mice, followed by apyrase or normal saline treatment after 2 weeks of inoculation. After the xenograft models successfully established, mice were randomly divided into different groups (with or without cisplatin) to investigate tumor growth and metastasis under cisplatin treatment. **B** Primary tumor size was measured (upper) and quantified every 5 days (lower). **C** Primary tumors and representative metastasis specimens were HE stained (left). Necrosis area ratios in primary tumor and numbers of metastatic lesions in lung and liver (yellow arrowheads) were quantified (right). **D, E** Expressions of HIF-1α and its target genes from inoculated tumor tissue were detected via qRT-PCR (**D**) and western blotting (**E**). **F** Expressions of HIF-1α and its target genes as well as Ki-67 and cleaved caspase-3 were immunohistochemistry (IHC) stained. In IHC staining analysis, data were calculated by Image Pro-Plus (IPP) (Media Cybernetics, Inc., Rockville, MD, USA). Error bars represent means ± SD from triplicates. Data are representative of at least three independent experiments. *p < 0.05, **p < 0.01, ***p < 0.001; ns, not significant.

To test the ability of ATP-HIF-1α signaling in mediating chemoresistance in vivo, 10^6^ stably transfected MDA-MB-231 cells were injected into the mammary fat pad of female BALB/c nude mice (Fig. 4A). Initially, the mice were randomly divided into three groups (*n* > 12 each). One group was injected with MDA-MB-231 shNC cells followed by normal saline treatment (intraperitoneal injection, twice per week), one group was injected with MDA-MB-231 shNC cells followed by apyrase (an ATP hydrolase, 400 U/kg, intraperitoneal injection, twice per week), and one group was injected with MDA-MB-231 shHIF-1α cells followed by normal saline (intraperitoneal injection, twice per week). Two weeks after inoculation, when the tumor volumes reached ~200 mm^3^, we examined the expressions of HIF-1α and the HIF-1 target genes *ADM* and *PDK1* in the apyrase and shHIF-1α groups (Fig. S2) to verify the successful establishment of the xenograft models. Mice in each group were further randomly divided into two groups (*n* = 6 each), and one group was treated with cisplatin (5 mg/kg, intraperitoneal injection, twice per week) for 3 weeks. Mice were sacrificed after a total of 5 weeks (Fig. 4A). Tumor volume was measured every 2 days and quantified every 5 days, as shown in Fig. 4B. We found that apyrase, shHIF-1α, and cisplatin treatment were associated with a significant decrease in primary tumor volume (Fig. 4B) and metastatic lesions in the lung and liver (Fig. 4C), indicating a less malignant phenotype.

In particular, we found that mice in the shNC+apyrase+cisplatin and shHIF-1α + cisplatin groups had smaller tumor volumes (Fig. 4B) and decreased invasion into the neighboring tissues (Fig. 4C). We also found decreased necrosis and tumor area ratio as well as fewer metastatic lesions in the liver and lung (Fig. 4C) compared with cisplatin treatment alone. This indicates that apyrase treatment and HIF-1α-knockdown made the tumors more sensitive to cisplatin. Downregulations of HIF-1α and the HIF-1 target genes *ADM* and *PDK1* in the apyrase-treated and shHIF-1α knockdown groups with or without cisplatin were observed by qRT-PCR (Fig. 4D), western blotting (Fig. 4E), and immunohistochemistry (IHC) staining (Fig. 4F) of inoculated tumors. These in vivo findings are in agreement with the initial in vitro results. In addition, the level of Ki-67 was decreased, and cleaved caspase-3 was increased in shNC+apyrase+cisplatin and shHIF-1α + cisplatin groups (Fig. 4E). These results support the finding that these treatments make cancer cells more sensitive to cisplatin treatment.

These results demonstrate the significant effect of HIF-1α-knockdown in cisplatin treatment using HIF-1α shRNA. However, it is difficult to disrupt endogenous expression in vivo, but direct HIF inhibitors have been identified that target HIF expression and/or function by various mechanisms [22]. To pursue this therapy in a pre-clinical application, we treated mice with 2-Methoxyestradiol (2-MeOE2), which is an inhibitor of HIF-alpha protein synthesis and transcriptional activity that is currently involved in Phase II clinical trials [20, 22]. Female BALB/c mice were injected with 10^6^ MDA-MB-231 cells into the mammary fat pad. Two weeks after tumor cell inoculation, the mean tumor volume was ~200 mm^3^ and we began treating cohorts of mice (*n* = 6 per group) with: (a) normal saline, (b) 2-MeOE2 (20 mg/kg, orally, every day), (c) cisplatin (5 mg/kg, intraperitoneal injection, twice per week), or (d) a combination of 2-MeOE2 and cisplatin for 3 weeks. The mice treated with the combination therapy demonstrated significant growth inhibition and decreased metastasis without affecting body weight, suggesting the potential application of inhibiting HIF function with 2-MeOE2 treatment in clinical breast cancer therapy (Fig. S3).

### ATP-HIF-1α signaling mediates spheroid formation in cisplatin treatment

**Fig. 5:**
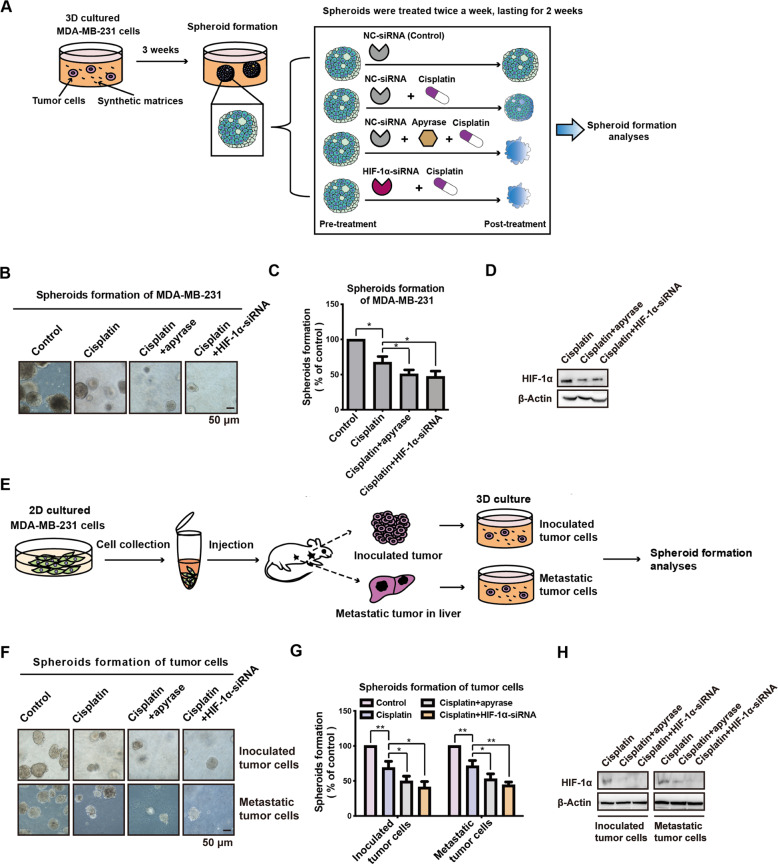
ATP-HIF-1α signaling mediates spheroid chemoresistance in vitro. **A** The schema chart for spheroid formation in 3D culture. MDA-MB-231 cells grown in spheroid media were used to generate spheroids for 3 weeks, and then treated with apyrase (0.2 U/ml) or HIF-1α-siRNA plus cisplatin (20 μM) for additional 2 weeks. And spheroid formation assay was used to test drug sensitivity. **B–D** The spheroids were photographed (**B**) and calculated compared with the control group (**C**). Knockdown efficiency of HIF-1α was demonstrated via western blotting (**D**). **E** Schema chart for primary culture of inoculated- and metastatic-tumor cells and spheroid formation assay. **F–H**. Inoculated- and metastatic-tumor cells were used for spheroid formation assay, along with apyrase (0.2 U/ml) or HIF-1α-siRNA, plus cisplatin (20 μM) treatment. The spheroids were photographed (**F**) and spheroid formation efficiency was calculated (**G**). Knockdown of HIF-1α was detected by western blotting (**H**). Error bars represent means ± SD from triplicates. Data are representative of at least three independent experiments. *p < 0.05, **p < 0.01, ***p < 0.001; ns, not significant.

To directly observe the effects of targeting extracellular ATP-HIF-1α signaling combined with cisplatin, spheroid formation assays were performed. MDA-MB-231 cells were used to generate spheroids for 3 weeks, and then treated with apyrase (0.2 U/mL) or HIF-1α-siRNA + cisplatin (20 μM) for an additional 2 weeks (Fig. 5A). Decreased spheroid formation capacities were observed in the apyrase and HIF-1α-siRNA groups by cell imaging (Fig. 5B, 5C). We observed decreases in HIF-1α by apyrase and HIF-1α-siRNA by western blotting (Fig. 5D).

To further investigate the function of ATP-HIF-1α signaling in mediating chemoresistance in both primary tumors and metastatic lesions, we performed primary cultures of tumor cells for the spheroid formation assay. Briefly, BALB/c mice were injected with 10^6^ MDA-MB-231 cells in the mammary fat pad (*n* = 6). After 1 month, we collected cancer cells from the inoculated tissue and liver metastatic lesions for three-dimensional (3D) culture (Fig. 5E). Tumor cells were treated with apyrase (0.2 U/mL) or HIF-1α-siRNA plus cisplatin (20 μM). We found that apyrase and HIF-1α-siRNA caused significant reductions in the spheroid formation capacity of these tumor cells as well as reduced the expression of HIF-1α (Fig. 5F–H), indicating that apyrase and HIF-1α-siRNA both enhanced cisplatin sensitivity in 3D tumor models.

### Co-targeting of STAT3 and AKT suppresses HIF-1α signaling and chemoresistance

The findings from the mouse and spheroid models demonstrated the potential of targeting the ATP-HIF-1α signaling pathway to increase drug sensitivity. However, HIF-1α protein is highly unstable under normoxic conditions and unstable in most tissues [36], making it difficult to target. Considering that targeting the upstream signaling of HIF-1α is also a potential strategy in cancer therapy [20], we aimed to investigate the effect of co-targeting of STAT3 and AKT, which are upstream of HIF-1α function. We used S3I-201 and LY294002 to inhibit the phosphorylation of STAT3 and PI3K/AKT, respectively.

**Fig. 6:**
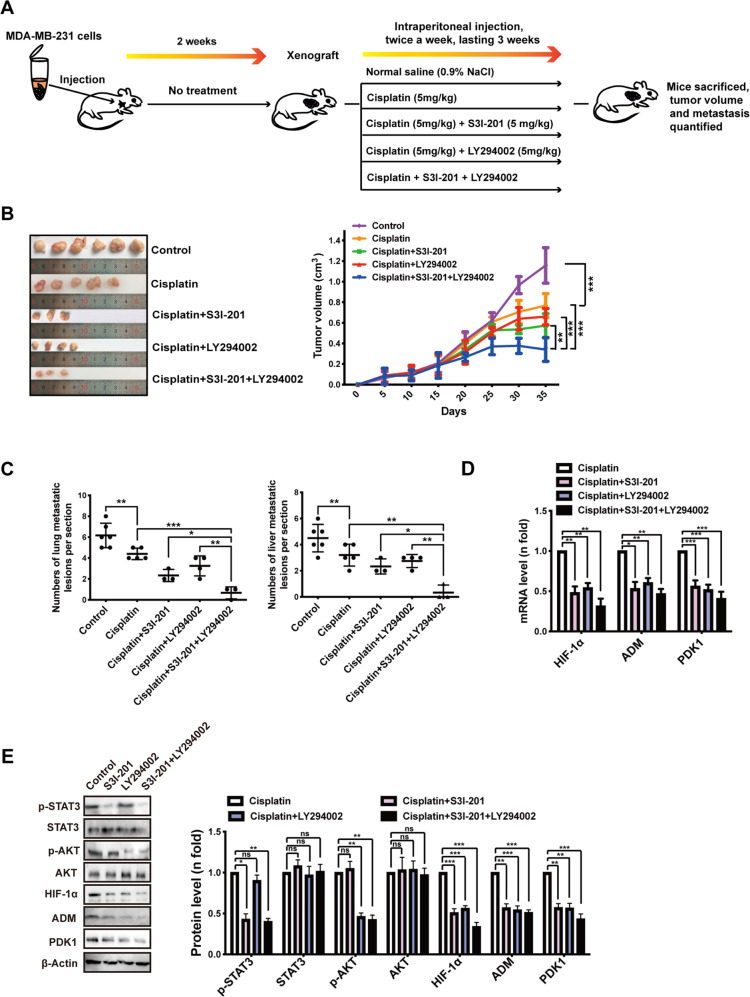
In vivo significance of co-targeting STAT3 and AKT to suppress HIF-1α signaling and to increase drug sensitivity. **A** A proposed model of xenograft experiment. In brief, six million MDA-MB-231 cells were implanted subcutaneously into the flanks of Balb/c nude mice. Dosing of the indicated drugs was initiated when tumor sizes reached 200 mm^3^ after 2 weeks. **B** Primary tumor size was measured (left) and quantified (right). **C** Primary tumor and representative metastasis specimens (yellow arrow heads) were HE stained (left). The numbers of metastatic lesions in lung and liver were quantified (right). **D** Expressions of HIF-1α and its target genes from inoculated tumor tissue were detected via qRT-PCR. **E** Western blotting illustrated the inhibition of STAT3 or PI3K/AKT signaling by its corresponding inhibitor as well as the downregulations of HIF-1α and its target genes. Error bars represent means ± SD from triplicates. Data are representative of at least three independent experiments. *p < 0.05, **p < 0.01, ***p < 0.001; ns, not significant.

To investigate the effect of co-targeting STAT3 and AKT in increasing drug sensitivity in vivo, 10^6^ MDA-MB-231 cells were injected into the mammary fat pad of female BALB/c nude mice. Two weeks after inoculation, the tumor volumes reached ~200 mm^3^ and mice were randomly divided into five groups (*n* = 6 each group) and treated with: (a) saline (denoted as control), (b) cisplatin (5 mg/kg), (c) cisplatin (5 mg/kg) plus S3I-201 (5 mg/kg), (d) cisplatin (5 mg/kg) plus LY294002 (5 mg/kg), or (e) cisplatin plus S3I-201 and LY294002 (both 5 mg/kg). Mice were treated twice per week via intraperitoneal injection for 3 weeks and then sacrificed a total of 5 weeks after inoculation, when the tumor size of the control group reached ~1,200 mm^3^ (Fig. 6A).

The S3I-201 and LY294002 combination group showed slower tumor growth than the control group or the groups treated with S3I-201 or LY294002 alone (Fig. 6B), while dual inhibition without cisplatin did not strongly reduce the tumor volume (Fig. S4). We also found a decrease in the number of metastatic lesions in the lung and liver in the combination treatment group (Fig. 6C), indicating greater drug sensitivity. It is worth noting that there was no significant difference in the weight of mice among the treatment groups, suggesting that there was no additional toxicity in the combination treatment.

At the molecular level, downregulations of HIF-1α and the HIF-1 target genes *ADM* and *PDK1* in S3I-201- and LY294002-treated groups were demonstrated by qRT-PCR (Fig. 6D) and western blotting (Fig. 6E) in inoculated tumors. The levels of phosphorylated STAT3 and AKT were also confirmed to be reduced in these tissues by western blotting (Fig. 6E), which are in line with our in vitro findings.

We conducted spheroid formation assays, which demonstrated the therapeutic effect of co-targeting STAT3 and AKT to inhibit HIF-1α signaling and decrease tumor growth in combination with cisplatin treatment (Fig. S5). Taken together, these studies shed light on combination drug treatment based on co-targeting upstream regulators of HIF-1α signaling.

### ATP-HIF-1α signaling is associated with clinical breast cancer progression

**Fig. 7:**
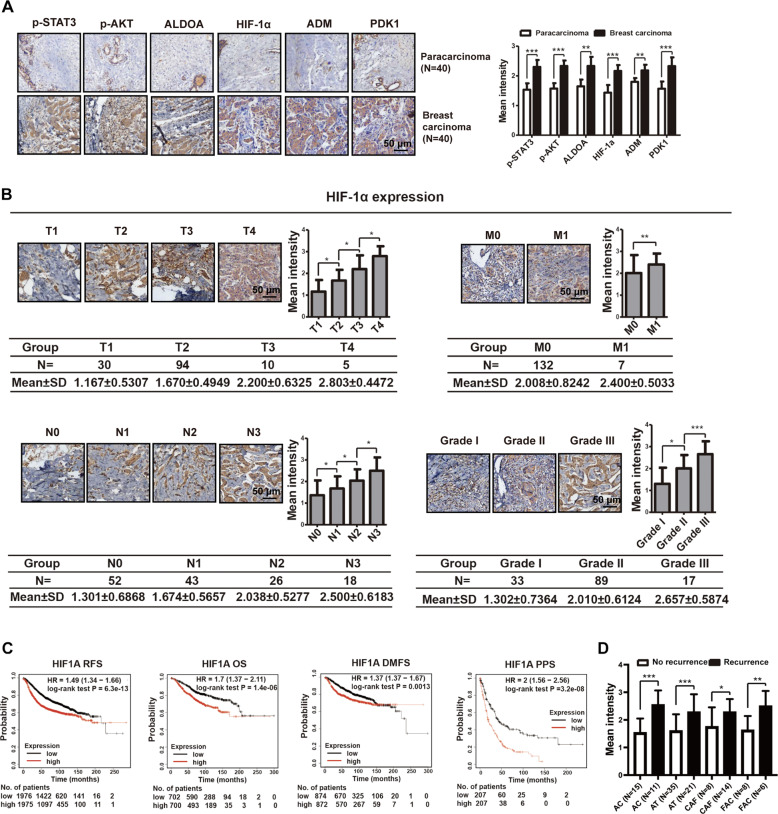
ATP-HIF-1α signaling is associated with clinical breast cancer progression. **A** IHC staining demonstrated high expressions of molecules involved in ATP-HIF-1α signaling in breast cancer tissues compared with paired paracarcinoma tissues. **B** IHC illustrated that HIF-1α level was associated with breast cancer TNM stage and grade. Data were calculated by Image Pro-Plus (IPP) (Media Cybernetics, Inc., Rockville, MD, USA). **C** Kaplan–Meier survival analysis (http://kmplot.com/analysis/) showed the negative correlations between survival time of breast cancer patients and expressions of HIF-1α. RFS relapse-free survival; OS overall survival; DMFS distant metastasis-free survival; PPS post-progression survival. **D** IHC showed that high expressions of HIF-1α is associated with recurrence after chemotherapy combination treatment. AC= Adriamycin + Cyclophosphamide, AT= Adriamycin + Taxotere, CAF= Cyclophosphamide + Adriamycin + Fluorouracil, FAC= Fluorouracil + Adriamycin + Cyclophosphamide. Error bars represent means ± SD from at least triplicates. Data are representative of at least three independent experiments. *p < 0.05, **p < 0.01, ***p < 0.001.

To investigate the role of HIF-1α in breast cancer progression in clinical settings, we analyzed the protein expressions of ATP-HIF-1α signaling-associated genes in 139 breast cancer specimens using tissue microarrays (TMAs), including 40 paired adjacent breast tissues, as described previously [14]. The expressions of ATP-HIF-1α signaling-related molecules were relatively higher in breast cancer tissues than in adjacent breast tissues (Fig. 7A). In particular, IHC staining showed that HIF-1α expression was positively associated with clinical TNM stages and grades of breast cancer (Fig. 7B). In addition, Kaplan–Meier survival analysis (http://kmplot.com/analysis/) [37, 38] was performed with the sources for the databases including GEO, EGA, and TCGA. In relapse-free survival (RFS), overall survival (OS), distant metastasis-free survival (DMFS), and post-progression survival (PPS) analyses, high mRNA levels of HIF-1α were associated with lower survival rates in patients with breast carcinoma (Fig. 7C). Because there were limited breast cancer specimens treated with chemotherapy available, we used another TMA that contained information about the patient chemotherapy and recurrence (Supplementary Table 2). We found that high levels of HIF-1α were also associated with recurrence after chemotherapy (Fig. 7D), suggesting drug resistance. Taken together, these data build upon our previous findings on the drug resistance of cancer cells.

## Discussion

The important role of extracellular ATP during cancer cell growth and invasion has been intensively investigated by many scientists, including our team. However, whether extracellular ATP is involved in chemoresistance and, if so, what the underlying mechanism remains unclear. We have previously explored different concentrations (0 μM, 50 μM, 100 μM, and 200 μM) of extracellular ATP to mediate resistance to chemotherapeutic drugs (cisplatin, doxorubicin, paclitaxel, gemcitabine) [14]. We found that 100 μM extracellular ATP showed maximal anti-apoptosis effect during drug treatment. We considered several possible explanations for this result. First, based on our previous research, 100 μM ATP stimulation showed maximal Ca^2+^ mobilization in cancer cells, causing dramatic induction of downstream signal transduction [39], in which genes that are involved in chemoresistance were significantly regulated, such as ATP-binding cassette subfamily B member 1 (*ABCB1*) and ATP-binding cassette subfamily G member 2 (*ABCG2*) [14, 40]. Second, 100 μM ATP is reported to promote tumor EMT, invasion, and metastasis [10, 12, 14], which is closely associated with reduced chemotherapy sensitivity [41–43]. Third, treatment with concentrations of ATP higher than 100 μM could arrest breast cancer cell proliferation, but 100 μM ATP had little effect on cellular viability [12]. The tumor microenvironment accumulation extracellular ATP that can reach hundreds of micromoles, and so 100 μM ATP is a pathological concentration and was applied for subsequent functional experiments.

The main reason for selecting *HIF1A* (encoding the HIF-1α protein) in the cDNA microarray for further study was that HIF-1α has shown great importance in guiding clinical cancer therapy. HIF expression can be used as a biomarker for response to treatment [22], displaying resistance to chemotherapy, radiotherapy, targeted therapy [22], and immunotherapy [44, 45]. Overexpression of HIF-1α has been reported in most carcinomas and indicates poor patient prognosis [22]. In addition, HIF-1α is a vital transcription factor that functions in tumor survival, proliferation, metabolism, invasion, metastasis, and angiogenesis [33]. Therefore, HIF-1α-associated signaling has been recognized as an important cancer drug target [20].

There are many subgroups of HIF-1 target genes that are particularly relevant to cancer which encode angiogenic factors, glucose transporters, glycolytic enzymes, survival factors, and invasion factors [20]. Evidence suggests that HIF-1 alone cannot account for this cell type-specific gene expression. Rather, it is the functional interaction of HIF-1 with other factors that determines the subgroup of HIF-1 target genes [27]. In this study, we used mass spectrometry analysis to elucidate the interaction between HIF-1α and ALDOA, and report the novel finding of regulation of HIF-1α by STAT3-ALDOA in ATP treatment. In addition, we demonstrate that *ADM* and *PDK1* function as two target genes of ATP-HIF-1-chemoresistance signaling. Evidence suggests that *ADM* could also function as a HIF-1 target gene to mediate angiogenesis and cell survival in solid tumors [32]. Kim et al. reported that HIF-1 could directly transactivate PDK1 and increase ATP levels to rescue cells from hypoxia-induced apoptosis [46]. These findings are in agreement with our findings that *ADM* and *PDK1* enhanced the resistance of cancer cells against apoptosis.

HIF-1-targeted therapeutics have shown efficacy in clinical cancer treatment [22]. HIF inhibitors have been studied as a single agent or in combination with other agents mainly for the treatment of advanced or refractory cancers [22]. Despite multiple direct HIF inhibitors being in Phase II and III clinical trials, there are few HIF inhibitors being tested in breast cancer therapies. In this study, we demonstrate the chemoresistance role of ATP-HIF-1α signaling both in vitro and in vivo by using conventionally cultured cell lines, 3D sphere formation assays, and BALB/c mouse models, suggesting the important role of HIF-1α in breast cancer therapy. However, it is clear that the HIF-1α regulation pathway is a highly complex network involving several signaling cascades and overlapping mechanisms. In addition, the HIF-1α protein is highly unstable under normoxic conditions in most tissues, making it difficult to target [36]. Therefore, it is difficult to develop specific HIF-1α inhibitors [32]. As HIF-1α signaling could be regulated by ATP-associated signaling, we combined S3I-201 and LY294002 to co-target the upstream signaling components of the ATP-HIF-1α signaling pathway, and our in vivo mouse model showed increased drug sensitivity of cancer cells to chemotherapy. This combination method has potential to improve the current clinical breast cancer therapies.

There are two main limitations of this study. First, in the clinical setting, there are different strategies that are employed for triple-negative breast cancer and ER-positive breast cancer [47]. We mainly focused on the general activation of HIF-1α signaling by ATP in MDA-MB-231 and MCF-7 cells, providing a relatively broad array of targets for breast cancer therapy. However, whether triple-negative breast cancer cells and ER-positive cells function through the same effectors still need to be explored. Another limitation is that different treatment timings were not involved in dual or triple therapy (i.e., all therapies were given together at the same time). It is possible that delivering the inhibitors sequentially would be more effective in breast cancer therapy, which should be investigated in the future.

**Fig. 8:**
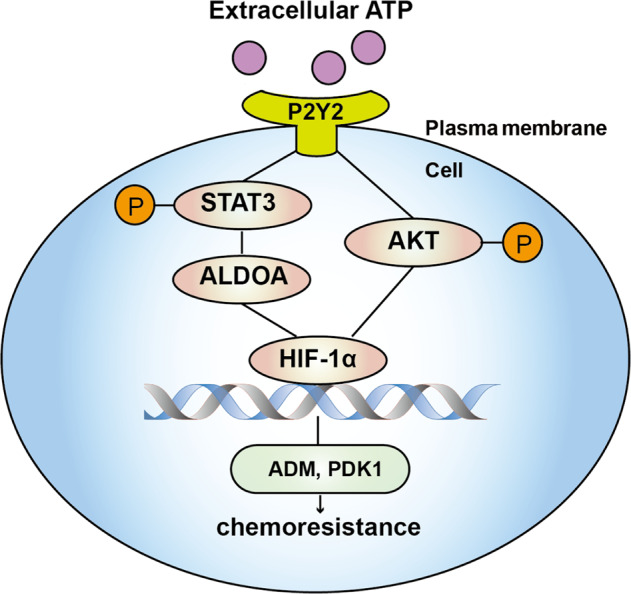
A proposed model of the ATP-HIF-1α signaling. Extracellular ATP could stimulate STAT3-ALDOA and AKT signaling via P2Y2 receptor and then regulate HIF-1α as well as HIF-1α target genes, among which ADM and PDK1 mediate ATP-driven chemoresistance.

In summary, we report the novel finding that HIF-1α can be elevated *via* extracellular ATP signaling in an O_2_-independent manner. We revealed the roles of ATP-HIF-1α and the HIF-1 target genes *ADM* and *PDK1* in promoting breast cancer chemoresistance. Additionally, HIF-1α can be regulated by both STAT3 and AKT in ATP signaling (Fig. 8). These findings have significant implications for our understanding of breast cancer progression. The pleiotropic effects of ATP-HIF-1α signaling in chemoresistance suggest that this signaling could be an effective target for breast cancer therapy.

## Materials and methods

### Cell lines and cell culture

The human breast cancer cell line MDA-MB-231 and normal breast cell line MCF10A were obtained from the American Type Culture Collection (ATCC, Manassas, VA, USA). MCF-7 cells were purchased from the Cell Resource Center, Institute of Basic Medical Sciences, Chinese Academy of Medical Sciences (Beijing, China). The culture medium has been described in our previous study [14]. The cells were cultured at 37 °C in a humidified CO_2_ incubator (5% CO_2_, 95% air). Cell lines were validated for authentication using the short tandem repeat (STR) method. All cell lines were determined to be free of mycoplasma contamination using the Mycoplasma PCR Detection Kit (Sigma, St. Louis, MO, USA).

### Spheroid formation assay

For tumor cell culture and spheroid formation, MDA-MB-231 cells or tumor cells were counted, resuspended, and plated at 10^3^ cells per well in a six-well plate. The medium contained DMEM/F12 (Sigma Aldrich, St Louis MO, USA) supplemented with B27 (Gibco Grand Island, NY, USA), 20 ng/mL epidermal growth factor (Peprotech, Rocky Hill, NJ), 20 ng/mL basic fibroblast growth factor (Peprotech, Rocky Hill, NJ), and 10 ng/mL hepatocyte growth factor (Peprotech, Rocky Hill, NJ); 1% methylcellulose was added to prevent cell aggregation. After 3 weeks, spheroids were treated (twice per week) with apyrase (0.2 U/mL), siRNAs, S3I-201(50 μM), or LY294002 (50 μM) to evaluate drug sensitivity. The number of spheroids within each well was counted under a microscope (Olympus BX-51, Olympus, Hamburg, Germany), and images of representative fields were captured. Ten individual lesions were assessed under a bright microscope for each plate.

### Xenograft tumorigenesis assays

Female BALB/c mice aged 6 weeks were purchased from the Center of Experimental Animals (Peking University, Beijing, China) and bred under specific pathogen-free conditions. The mice were randomly assigned to groups. All experiments were approved by the Institutional Animal Care and Use Committee of Peking University (no. LA2014229).

To test the ability of ATP-HIF-1α signaling to mediate chemoresistance using the shHIF-1α method, 10^6^ stably transfected MDA-MB-231 cells were injected into the mouse mammary fat pad. The mice were randomly divided into three groups (*n* > 12 each). One group was injected with MDA-MB-231 shNC cells followed by normal saline treatment, one group was injected with MDA-MB-231 shNC cells followed by apyrase, and one group was injected with MDA-MB-231 shHIF-1α cells followed by normal saline treatment. Two weeks after inoculation, when the tumor volumes reached ~200 mm^3^, mice in each group were further randomly divided into two groups (*n* = 6 each). One group was treated with cisplatin and mice were observed for three more weeks.

All mice were sacrificed after a total of 5 weeks. Tumor volume was measured every 2 days and quantified every 5 days. Tumor tissues were collected for hematoxylin and eosin (H&E), IHC, and western blotting analyses. Investigators were blinded to the group allocation when assessing the results.

Other xenograft tumorigenesis assays, including direct HIF-1α inhibitor, S3I-201, and LY294002 in tumor growth, are suppled in supplementary methods.

### Statistical analysis

For all analyses, results are presented as the mean ± standard deviation (SD) in the histograms unless otherwise noted. Student’s *t*-test, two-way ANOVA, or one-way ANOVA with Dunnett’s multiple comparisons test were used to determine the differences between groups. We statistically compared the similar variances between the groups as well. All experiments were repeated at least three times. The data were analyzed using the software package SPSS 20.0 (SPSS Inc., Chicago, IL, USA). Statistical significance was set at *P* < 0.05.

Details of additional methodologies can be found in supplementary materials and methods.

## Supplementary information

Supplementary Figures

Supplementary Material and Methods

Original Data File

Supplementary Table 1

Supplementary Table 2

Reproducibility checklist accompanied this article

## Data Availability

The cDNA microarray data of MCF-7 cells treated with extracellular ATP was provided in the GEO database (accession number: GSE113757). The rest datasets used or analyzed during the current study are available from the corresponding author on reasonable request.
