# Supplementary material for: Extracellular ATP promotes breast cancer chemoresistance via HIF-1α signaling
Source: Cell Death Dis. 2022 Mar 2;13(3):199. doi: 10.1038/s41419-022-04647-6 (PMC8891368; doi:10.1038/s41419-022-04647-6)
Supplement: Supplementary file 1 — Supplementary Figures [file 41419_2022_4647_MOESM1_ESM.docx]

**Supplementary Figures**


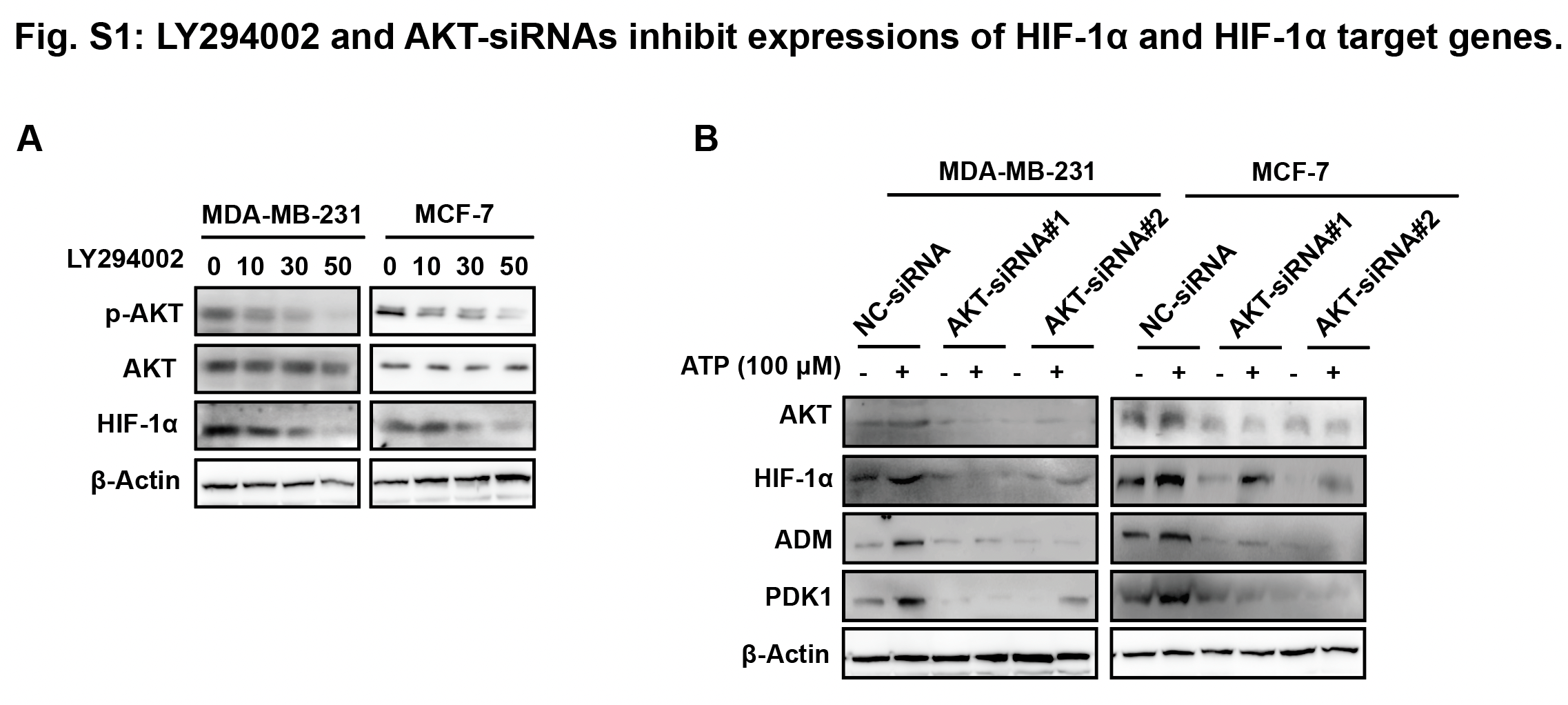


**Figure S1. LY294002 and AKT-siRNAs inhibit expressions of HIF-1α and HIF-1α target genes.** A. Western blotting illustrated alterations of p-AKT and HIF-1α in different dose of LY294002. B. Western blotting demonstrated the knockdown of AKT and the decreases of HIF-1α and its target genes.


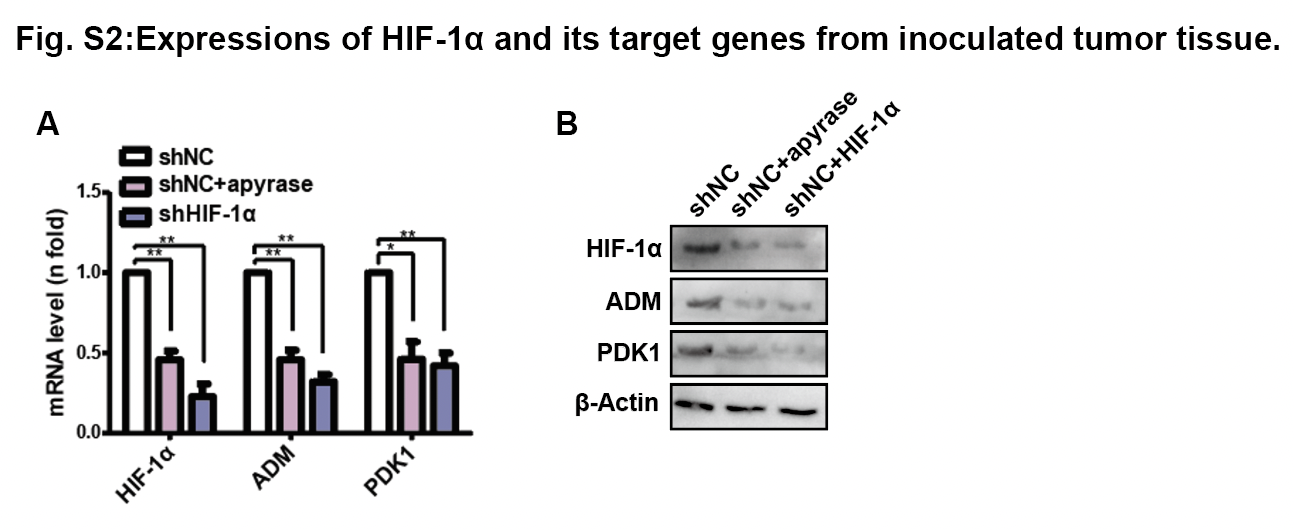


**Figure S2. Expressions of HIF-1α and its target genes from inoculated tumor tissue. A**. qRT-PCR and **B**. Western blotting demonstrated that expressions of HIF-1α and its target genes were down-regulated by apyrase or shHIF-1α after two weeks of inoculation. Error bars represent means ± SD from triplicates. Data are representative of at least three independent experiments. *, p < 0.05, **, p < 0.01.

**
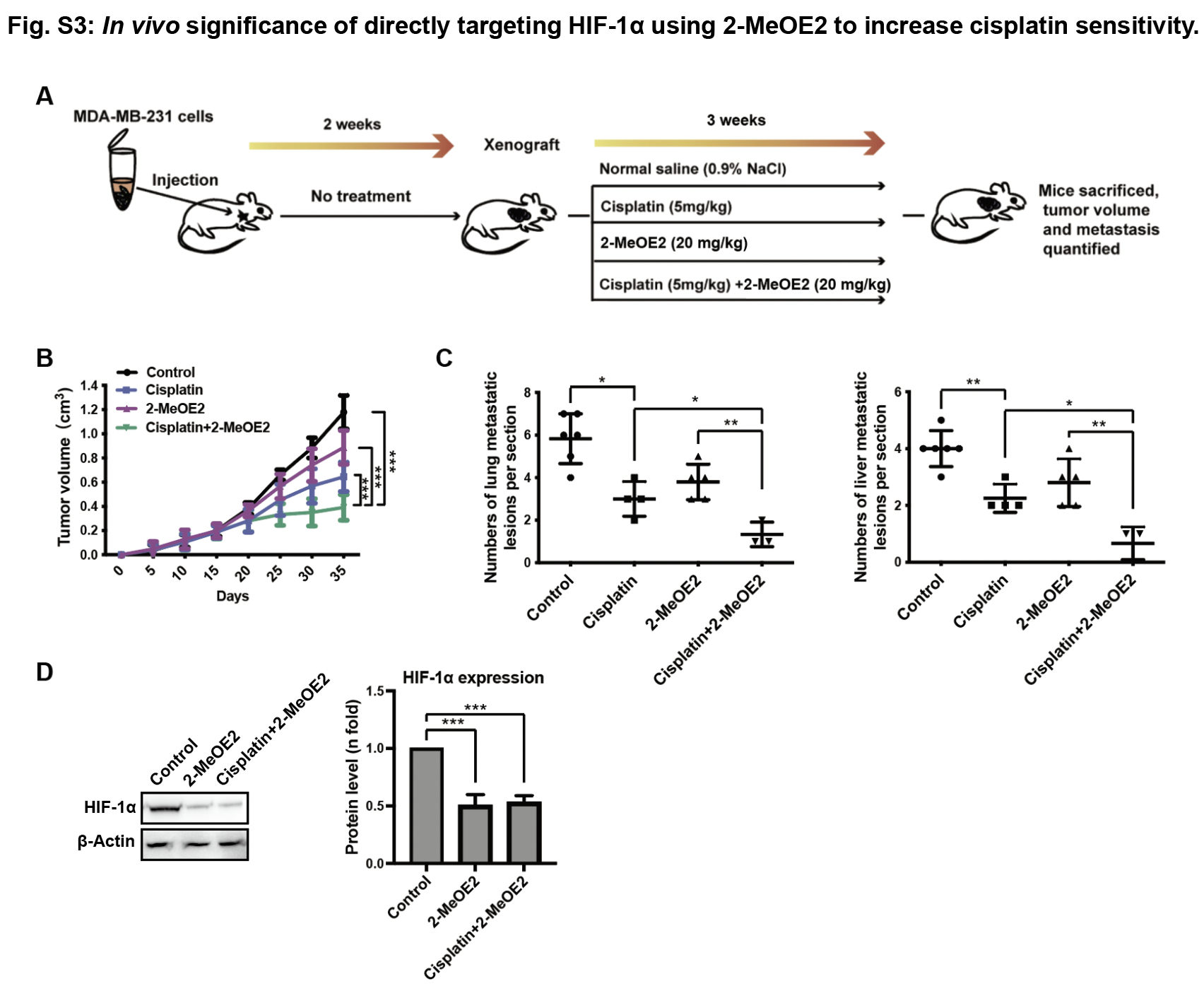
**

**Figure S3. *In vivo* significance of directly targeting HIF-1α using 2-MeOE2 to increase cisplatin sensitivity. A**. A proposed model of xenograft experiment. In brief, six million MDA-MB-231 cells were implanted subcutaneously into the flanks of BALB /c nude mice. Mice were randomly derived into four groups when tumor sizes reached 200 mm^3^ after two weeks and treated with normal saline, 2-MeOE2 (20 mg/kg, orally every day), cisplatin (5mg/kg, intraperitoneal injection, twice per week), or a combination of 2-MeOE2 and cisplatin respectively for another three weeks. And then mice were sacrificed for further analysis. **B**. Primary tumor size was measured and quantified in the five weeks. **C**. Numbers of metastatic lesions in lung and liver were quantified according to HE staining. **D**. Western blotting demonstrated the inhibition of HIF-1α by 2-MeOE2 in 2-MeOE2 treated group and combination treated group. Error bars represent means ± SD from triplicates. Data are representative of at least three independent experiments. *, p < 0.05, **, p < 0.01, ***, p < 0.001.

**
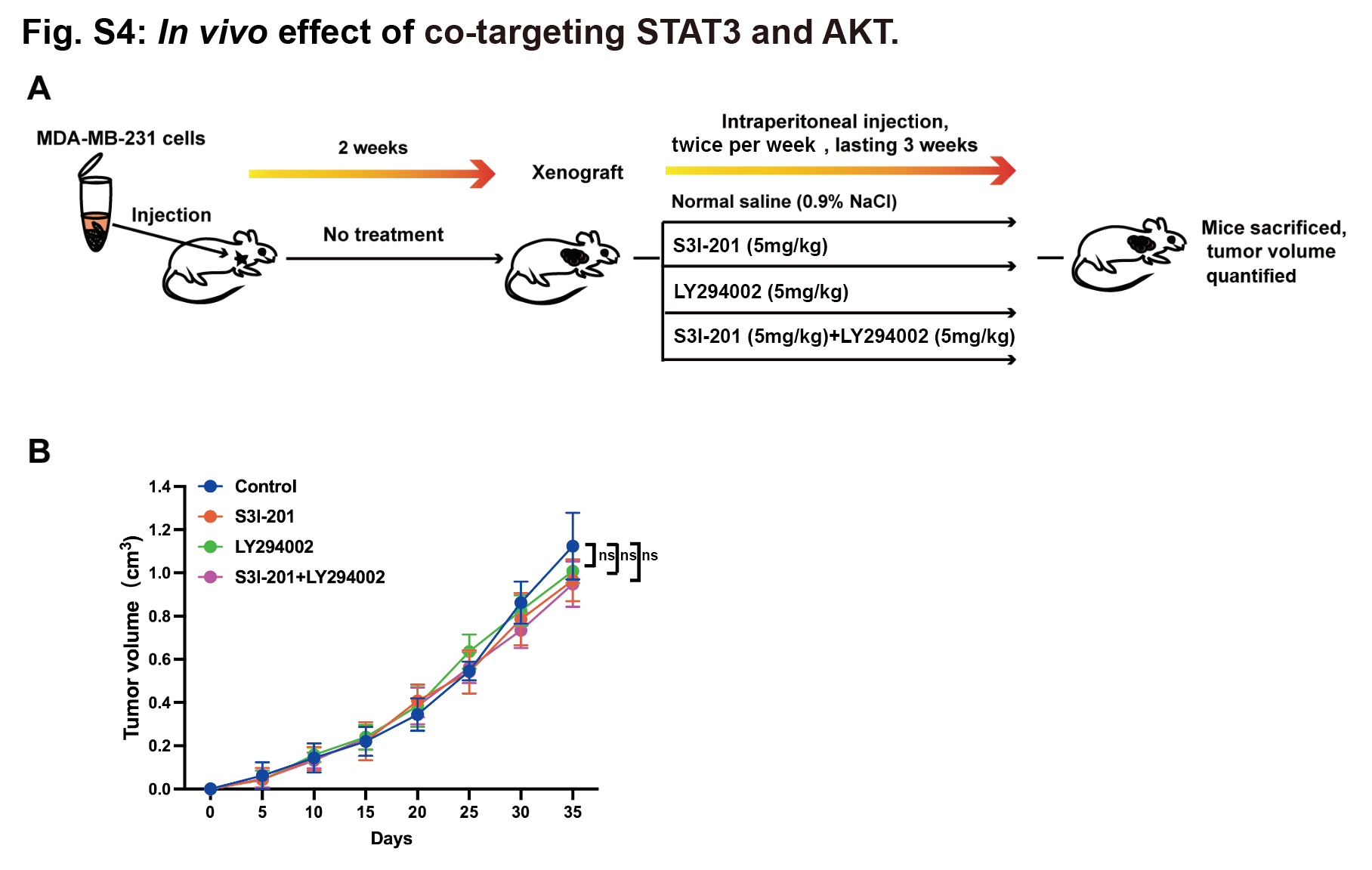
**

**Figure S4. *In vivo* effect of co-targeting STAT3 and AKT. A**. A proposed model of xenograft experiment. In brief, six million MDA-MB-231 cells were implanted subcutaneously into the flanks of BALB/c nude mice. Mice were randomly derived into four groups (n = 5 each) when tumor sizes reached 200 mm^3^ after two weeks, and treated twice per week with (a) saline (denoted as control), (b) S3I-201 (5 mg/kg), (c) LY294002 (5 mg/kg), or (d) S3I-201 plus LY294002 (both 5 mg/kg) for another three weeks. And then mice were sacrificed for further analysis. **B**. Primary tumor size was measured and quantified in the five weeks. Error bars represent means ± SD from triplicates. ns, not significant.


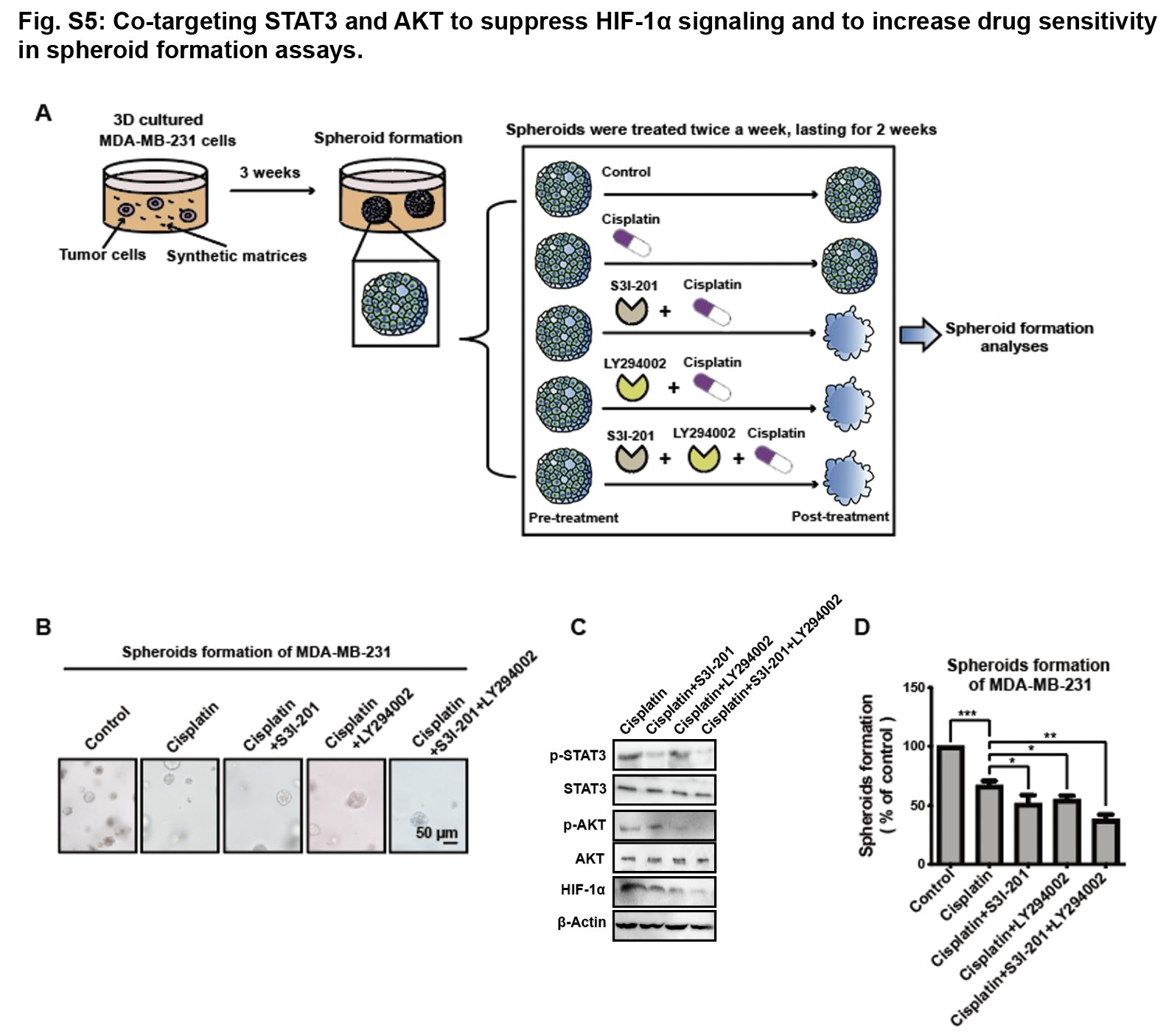


**Figure S5. Co-targeting STAT3 and AKT to suppress HIF-1α signaling and to increase drug sensitivity in spheroid formation assays. A**. The schema chart for spheroid formation in 3D culture. MDA-MB-231 cells grown in spheroid media were used to generate spheroids in three weeks, and then treated (twice a week) with S3I-201(50 μM), or LY294002 (50 μM), or S3I-201 and LY294002 combination, plus cisplatin (20 μM) for additional two weeks. And spheroid formation assay was used to test drug sensitivity. **B-D**. The spheroids were photographed (B) and calculated compared with control group (C). The inhibition of STAT3 signaling, AKT signaling and down-regulated efficiency of HIF-1α was demonstrated via western blotting (D). Error bars represent means ± SD from triplicates. Data are representative of at least three independent experiments. *, p < 0.05, **, p < 0.01, ***, p < 0.001.
