# Supplementary material for: Extracellular ATP promotes breast cancer chemoresistance via HIF-1α signaling
Source: Cell Death Dis. 2022 Mar 2;13(3):199. doi: 10.1038/s41419-022-04647-6 (PMC8891368; doi:10.1038/s41419-022-04647-6)
Supplement: Supplementary file 2 — Supplementary Material and Methods [file 41419_2022_4647_MOESM2_ESM.doc]

**Supplementary Material and Methods**

**Antibodies and Reagents**

The antibodies used are listed as follows: HIF-1α (Cat#ab51608), phospho-STAT3 (Tyr 705, Cat#ab76315), STAT3 (Cat#ab32500), phospho-AKT (Thr 308, Cat# ab38449), AKT (Cat#ab18785), ADM (Cat#ab69117), PDK1 (Cat# ab110025), Ki-67 (Cat#ab15580) and cleaved caspase-3 (Cat#ab2302) were from Abcam (Cambridge, UK). And β-Actin (Cat#sc-8432), ALDOA (Cat#sc-390733) and P2Y2 (Cat#sc-518019) were from Santa Cruz Biotechnology (Santa Cruz, CA, USA). ATP was bought from Sigma (St Louis, MO, USA). Scrambled control siRNA (NC) and siRNAs against HIF-1α, ALDOA, ADM, PDK1 and P2Y2, scrambled control shRNA (shNC) and shRNA against HIF-1α (shHIF-1α) were obtained from Shanghai GenePharma Co., Ltd. (Shanghai, China). Protein A/G Sepharose CL-4B beads were purchased from Amersham Biosciences (Piscataway, NJ, USA), and protease inhibitor cocktail was from Roche Applied Science (Indianapolis, IN, USA). Small molecular inhibitors S3I-201 (STAT3 inhibitor), LY294002 (PI3K/AKT inhibitor) and 2-MeOE2 (HIF-1α inhibitor) were bought from Selleck (Houston, TX, USA).

**Cell transfection**

For transient gene silencing, a scramble siRNA used as a negative control and two distinct siRNA oligonucleotides targeting HIF-1α, ALDOA, ADM, PDK1 and P2Y2 were used respectively. Cells were transfected with indicated siRNA using Lipofectamine RNAi Max (Invitrogen, Carlsbad, CA, USA) according to manufacturer’s instructions. Sequences of siRNAs were listed as follows.

The sequences of siRNAs

| Control-siRNA | UUCUCCGAACGUGUCACGU |
| --- | --- |
| HIF-1α-siRNA#1 | GAGGAAAAGGGAAAAUCUAUU |
| HIF-1α-siRNA#2 | GUGGUUGGAUCUAACACUA |
| ALDOA-siRNA#1 | GCCUUGCCUGUCAAGGAAA |
| ALDOA-siRNA#2 | GCGUUGUGUGCUGAAGAUU |
| ADM-siRNA#1 | CCGAGUCUCUGUAUAAUCU |
| ADM-siRNA#2 | GCCAGAGCAUGAACAACUU |
| PDK1-siRNA#1 | AATCTTCTCAGGACACCATCCGTTCAA |
| PDK1-siRNA#2 | CGAACUCCUUUGAACUGGACUUACA |
| P2Y2-siRNA#1 | GUGCUAACAGUUGCCUUGA |
| P2Y2-siRNA#2 | GCCCAAGAGAUGAACAUCU |
| AKT-siRNA#1 | GAACAAUCCGAUUCACGUATT |
| AKT-siRNA#2 | UGACUUCGACUAUCUCAAATT |

HIF-1α shRNA was designed and purchased from Genepharma (Shanghai, China). Sequence targeting HIF-1α was inserted into pGPU6 RNAi plasmid to generate HIF-1α shRNA. A scramble sequence was inserted into pGPU6 RNAi plasmid and used as control shRNA (shNC). Cells were transfected with the shHIF-1α plasmid or shNC plasmid using Lipofectamine 2000 (Invitrogen, Carlsbad, CA). Stable transfected clones were selected by G418 (Gibco, Grand Island, NY).

**Quantitative real-time PCR**

Real-time PCR was performed as described by JL Zhang et al [1]. Gene expression levels were normalized to β-Actin. Relative expression level of mRNA was evaluated by using 2^-ΔΔCt^ method. The primer sequences for qRT-PCR analysis were listed as follows.

The primers for qRT-PCR analysis

| Gene | Strand | Sequence |
| --- | --- | --- |
| β-Actin | Forward | GGATGCAGAAGGAGATCACTG |
|  | Reverse | CGATCCACACGGAGTACTTG |
| HIF1A | Forward | CTCAAAGTCGGACAGCCTCA |
|  | Reverse | CCCTGCAGTAGGTTTCTGCT |
| ADM | Forward | CCGGGCTCGCTGACGTGAAG |
|  | Reverse | CCGGACTGCTGTCTTCGGGG |
| PDK1 | Forward | TCAACGACCACTTTGTCAAGCTCAGCT |
|  | Reverse | GGTGGTCCAGGGGTCTTAC |

**Immunofluorescence analysis**

Experiments were performed as described by Y Liu [2] with some modifications. Briefly, cells were incubated with anti-HIF-1α (1:200) or anti-ALDOA (1:200) antibody at 4°C overnight, followed by incubation with FITC-conjugated or Texas Red-conjugated IgG fluorescence secondary antibody (1:500 dilution; GIBIO, Beijing, China). Finally, cell nucleus was stained with DAPI (Vector Laboratories, Burlingame, CA, USA), and observed under a confocal microscope (TCS SP2 AOBS; Leica, Germany).

**Western blotting analysis**

Western blotting analysis was performed as described by JL Zhang et al [1]**.** Primary antibodies used: anti-HIF-1α (1:1000), anti-ALDOA (1:1000), anti-phospho-STAT3 (Tyr 705) (1:800), anti-STAT3 (1:800), anti-phospho-AKT (Thr308) (1:1000), anti-AKT (1:1000), anti-ADM (1:1000), anti-PGK1 (1:1000), anti-P2Y2 (1:500) anti-β-Actin (1:1000). Results were presented as the ratio of targeted proteins to β-Actin. Immunoreactive protein was visualized *via* chemiluminescence (Applygen Technologies Inc) and quantified *via* densitometry analysis, using ImageJ software (NIH Image, Bethesda, MD, USA). Uncropped bands are presented in Supplemental Material.

**Silver staining and mass spectrometry**

Silver staining and mass spectrometry assays were carried out as described by L Shan et al [3] using resultant materials from co-immunoprecipitation analysis. ALDOA enrichment from mass spectrometry was listed in Supplementary Table S1.

**Co-immunoprecipitation analysis**

Co-immunoprecipitation were carried out as described by L Shan et al [3]. In our research, primary antibody HIF-1α (1:200, 5μl) or ALDOA (1:200, 5μl) was used.

**GST pull-down assays**

GST, GST- HIF-1α, GST- ALDOA plasmids were used in this research. The GST vector, pGEX-4T-1 was purchased from GE healthcare. MDA-MB-231 cDNA (0.5 μg) obtained from RNA reverse transcription was used as PCR template to generate full-length coding sequences of HIF-1α and ALDOA genes. PCR products were digested by the restriction enzymes, purified on agarose gel, and extracted (Tiangen, China) prior to ligation into plentivector (T4 DNA ligase, Invitrogen).

GST-fused constructs were expressed in BL21 Escherichia coli. *In vitro* transcription and translation experiments were performed with rabbit reticulocyte lysate (TNT systems, Promega). The transcribed/translated products were bound to glutathione-sepharose beads *in vitro* in binding buffer (75 mM NaCl, 50 mM HEPES, pH7.9). Then, these products were washed off the beads, resuspended in 2×SDS-PAGE loading buffer, and analyzed by western blotting.

**Cell apoptosis assay**

Cells were exposed to corresponding chemotherapy drugs, ATP, inhibitors, or siRNAs for indicated time, and then harvested and resuspended in binding buffer.

After incubation with propidium iodide and FITC-labelled antibody against Annexin V following manufacturer’s protocol (Vazyme Biotech), at least 1×10^4^ cells per sample were analyzed with FACS Calibur (Becton Dickinson).

**CCK-8 assay**

Breast cancer cells were seeded in 96-well plates with 4×10^3^ cells/well density, 8 parallel wells were assigned to each group. After 0, 24, 48, 72 and 96 h of treatment with chemotherapy drugs, ATP or inhibitors, CCK-8 assays were performed according to the manufacturer’s instruction. OD450 values were detected on an ELISA plate reader.

**Trypan blue dye exclusion assay**

To measure cell counts, cells were stained with 0.4% Trypan blue solution (Quality Biotech) for 5 min. Ten individual samples were measured for group. The number of cells that took up Trypan blue were counted as dead cells using a hemocytometer, and expressed as a percentage of the total cell number.

**Colony formation assay**

Colony formation assay was performed to assess the effect of chemotherapy drugs with/without ATP on anchorage-independent growth. DMEM medium containing 0.6% agarose was poured into six-well culture plate to form the bottom layer. Cells treated (twice a week) with/without chemotherapy drugs or inhibitors or siRNAs were suspended in DMEM containing 0.3% agarose and plated on the top layer. Cells were plated in triplicate at the density of 0.5×10^3^ cells per well. Colony numbers were counted 21 days later. Ten individual lesions under bright microscope were calculated for each plate.

**Xenograft tumorigenesis assays**

To test the ability of ATP-HIF-1α signaling to mediate chemoresistance using a direct HIF-1α inhibitor, 10^6^ MDA-MB-231 cells were injected into the mouse mammary fat pad. Two weeks after inoculation, when the tumor volumes reached approximately 200 mm^3^, the mice were randomly divided into four groups (n = 6 each) and treated with saline, 2-MeOE2, cisplatin, or a combination of 2-MeOE2 and cisplatin for another three weeks.

To test the ability of S3I-201 and LY294002 to increase drug sensitivity, 10^6^ MDA-MB-231 cells were injected into the mouse mammary fat pad. Two weeks after inoculation, when the tumor volumes reached approximately 200 mm^3^, mice were randomly divided into five groups (n = 6 each) and treated with saline, cisplatin, cisplatin plus S3I-201, cisplatin plus LY294002, or cisplatin plus S3I-201 and LY294002 for three weeks.

At last, to test the ability of S3I-201 and LY294002 in tumor growth, 10^6^ MDA-MB-231 cells were injected into the mouse mammary fat pad. When the tumor volumes reached approximately 200 mm^3^ after 14 days, mice were randomly divided into four groups (n = 5 each) and treated with saline, S3I-201, LY294002, or S3I-201 plus LY294002 for three weeks.

All mice were sacrificed after a total of five weeks. Tumor volume was measured every two days and quantified every five days. Tumor tissues were collected for hematoxylin and eosin (H&E), IHC, and western blotting analyses. Investigators were blinded to the group allocation when assessing the results.

**Reference**

1. Zhang JL, Liu Y, Yang H, Zhang HQ, Tian XX, Fang WG: **ATP-P2Y2-beta-catenin axis promotes cell invasion in breast cancer cells.** *Cancer Sci* 2017, **108:**1318-1327.

2. Liu Y, Geng YH, Yang H, Yang H, Zhou YT, Zhang HQ, Tian XX, Fang WG: **Extracellular ATP drives breast cancer cell migration and metastasis via S100A4 production by cancer cells and fibroblasts.** *Cancer Lett* 2018, **430:**1-10.

3. Shan L, Zhou X, Liu X, Wang Y, Su D, Hou Y, Yu N, Yang C, Liu B, Gao J, et al: **FOXK2 Elicits Massive Transcription Repression and Suppresses the Hypoxic Response and Breast Cancer Carcinogenesis.** *Cancer Cell* 2016, **30:**708-722.
