# Supplementary material for: Extracellular ATP promotes breast cancer chemoresistance via HIF-1α signaling
Source: Cell Death Dis. 2022 Mar 2;13(3):199. doi: 10.1038/s41419-022-04647-6 (PMC8891368; doi:10.1038/s41419-022-04647-6)

**Whole immunoblots with molecular makers of each result**

We retain the positions of at least two molecular weight markers both above and below the band of interest. Full scans or images of uncropped blots are provided as follows.

**Uncropped blots related to Figure 1**


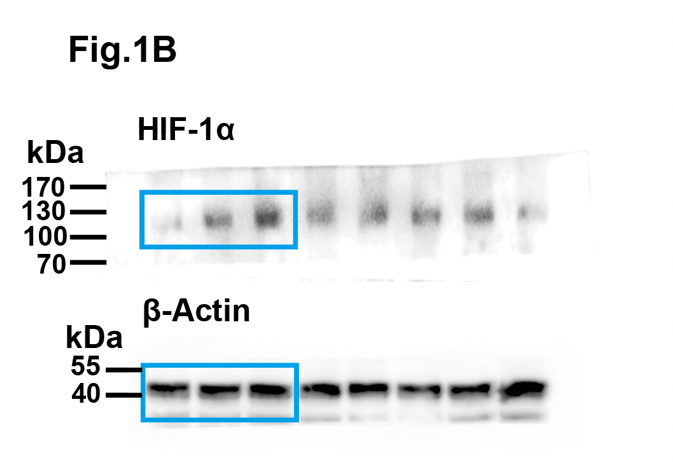


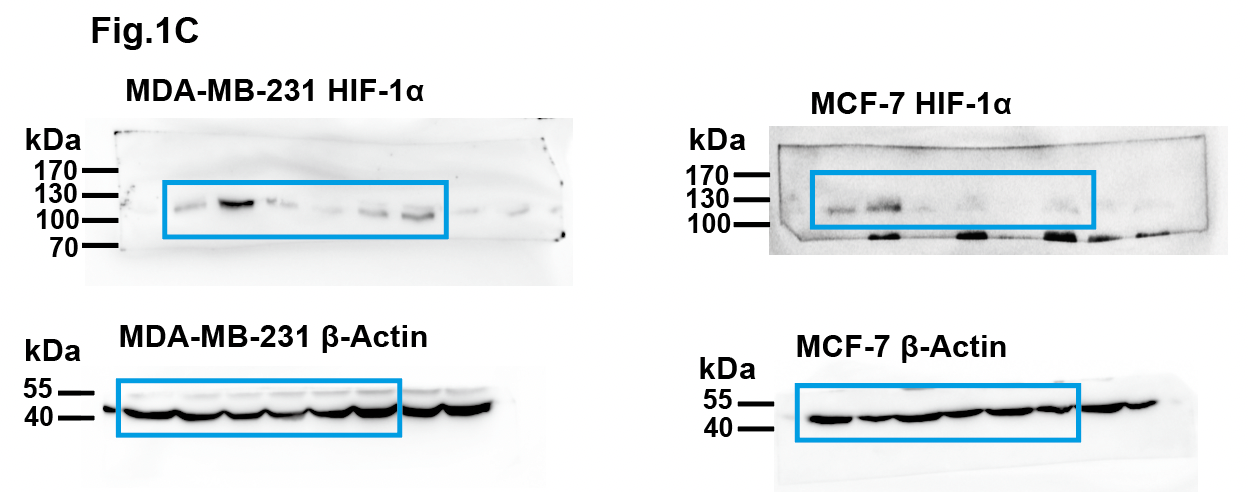


**Uncropped blots related to Figure 2**


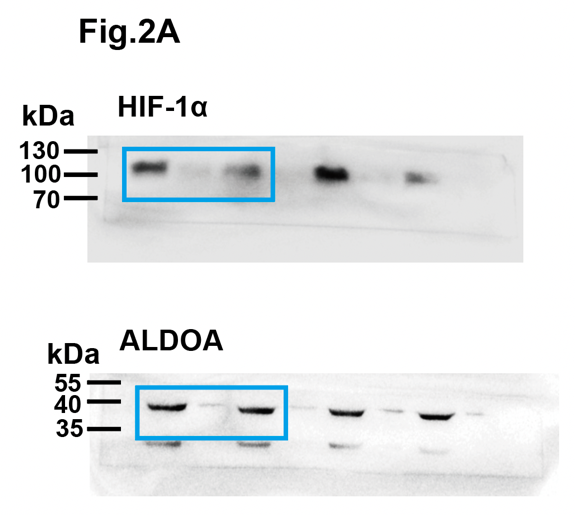


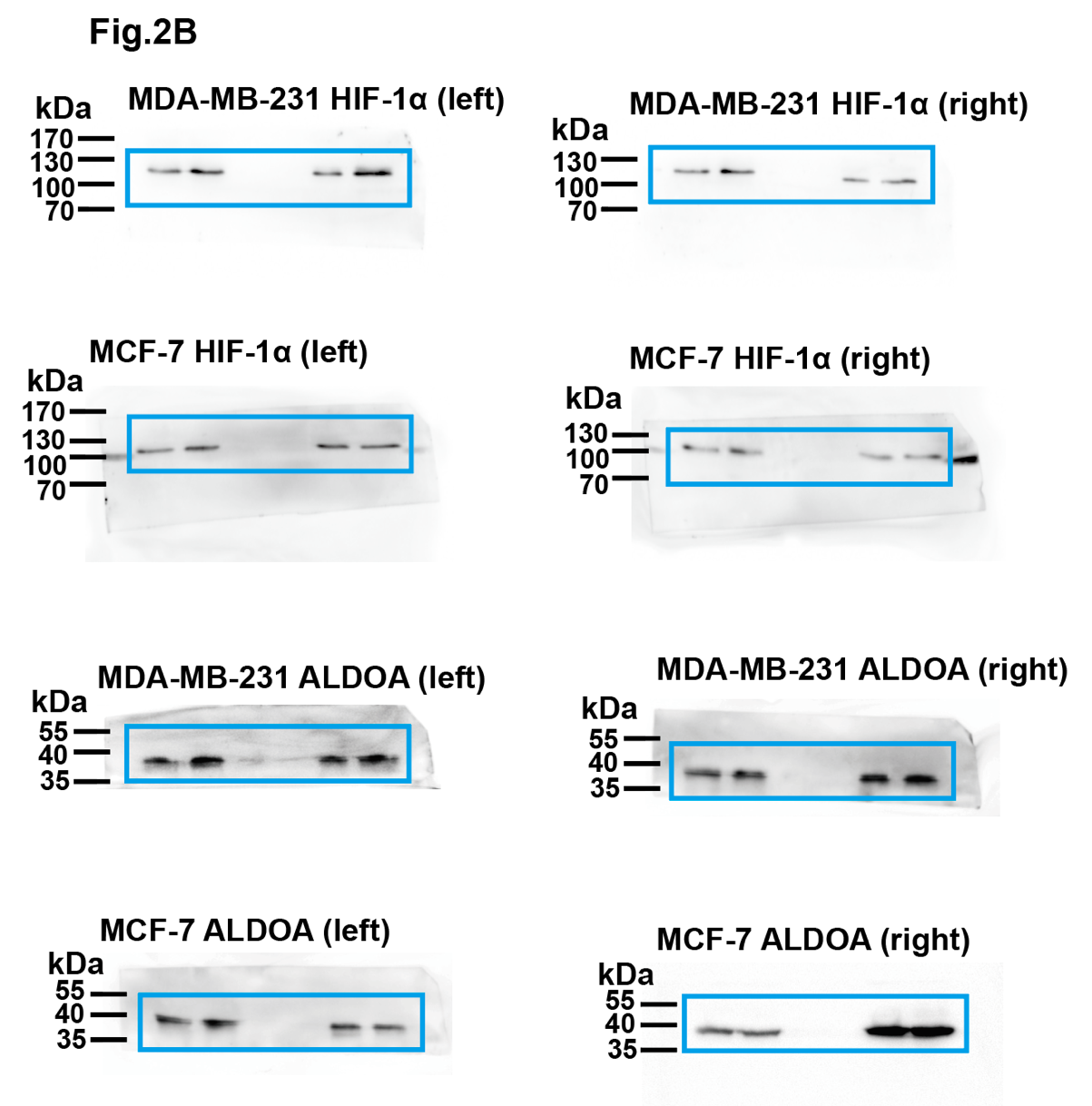


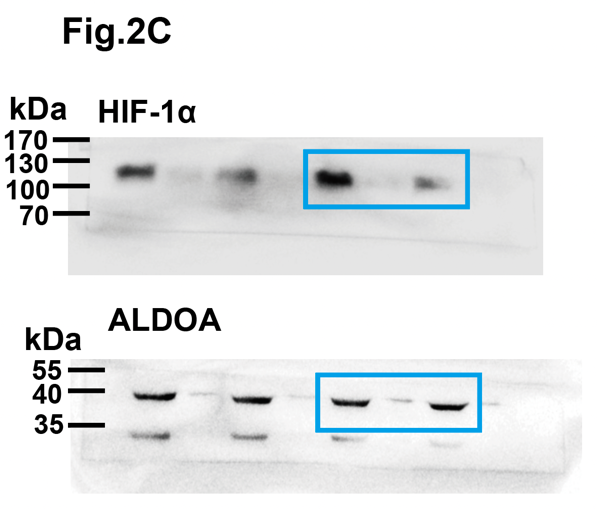


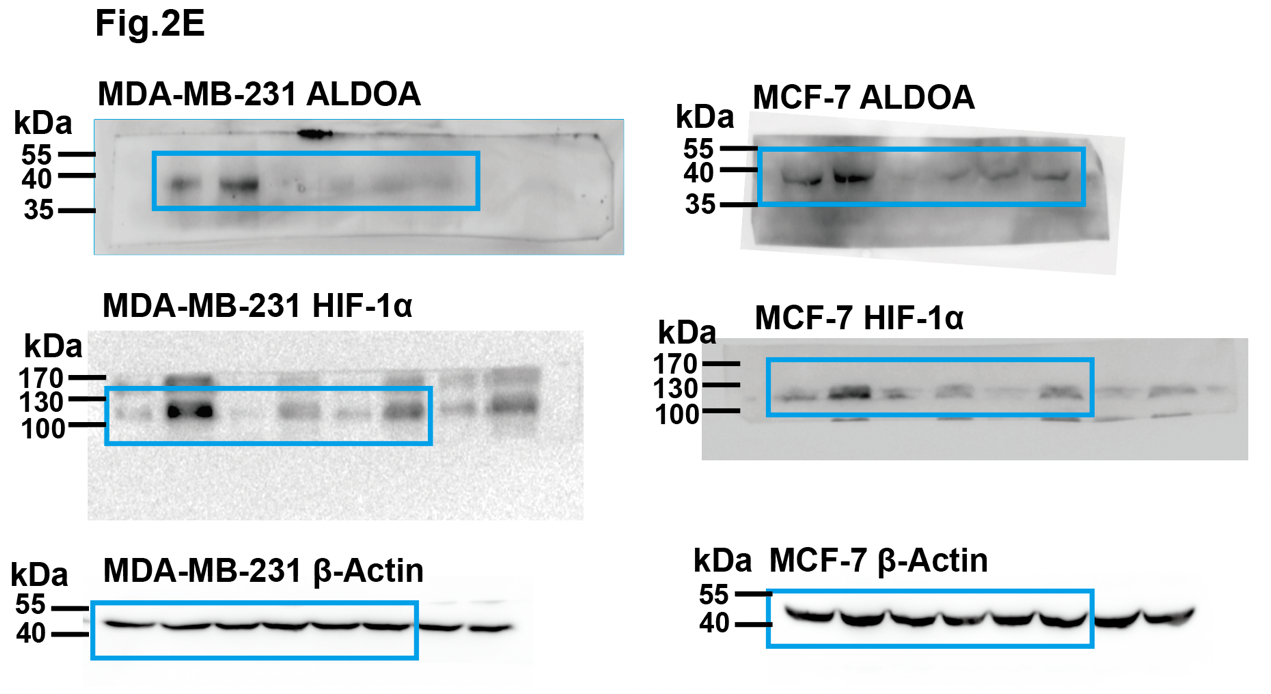


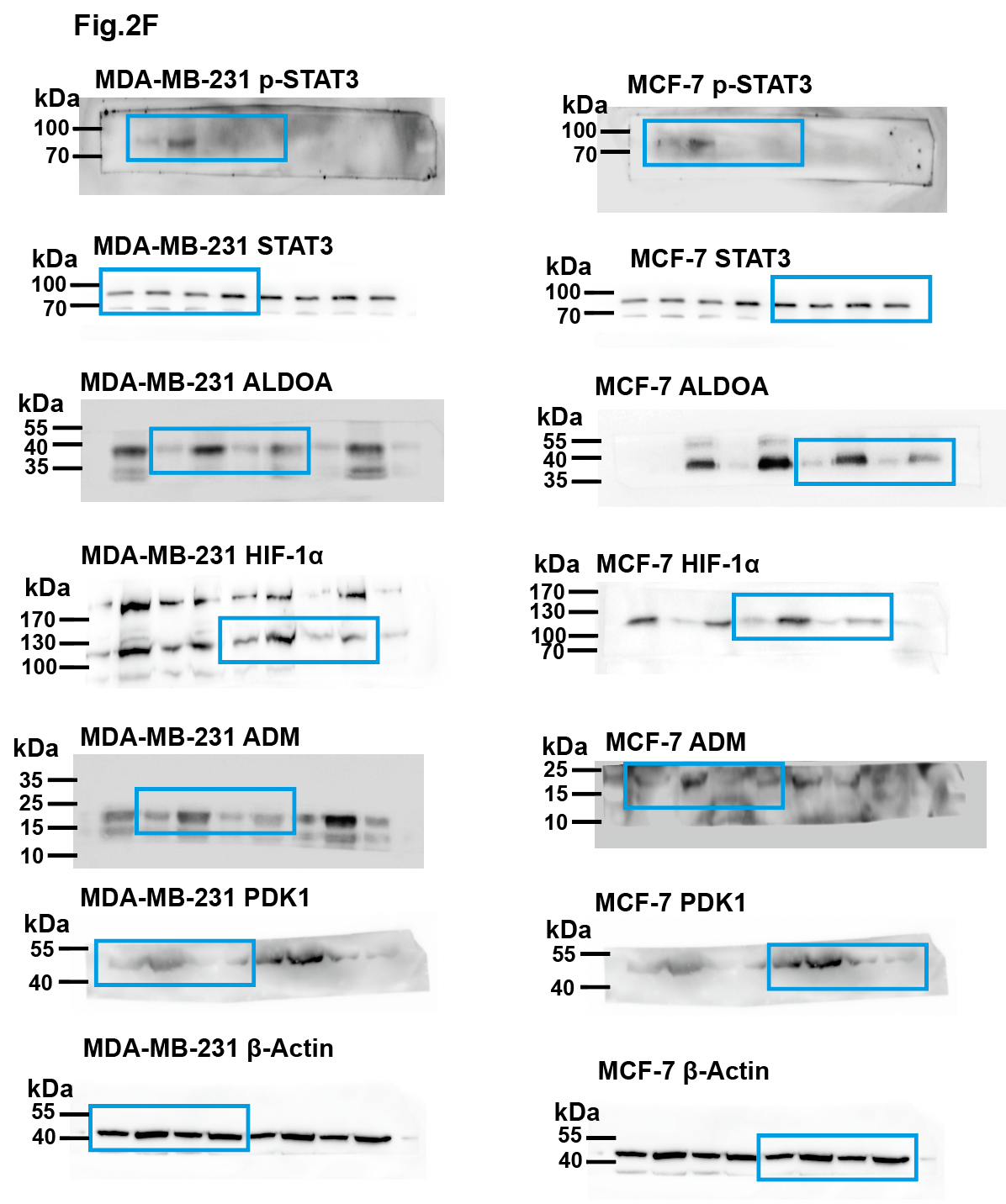


**Uncropped blots related to Figure 3**


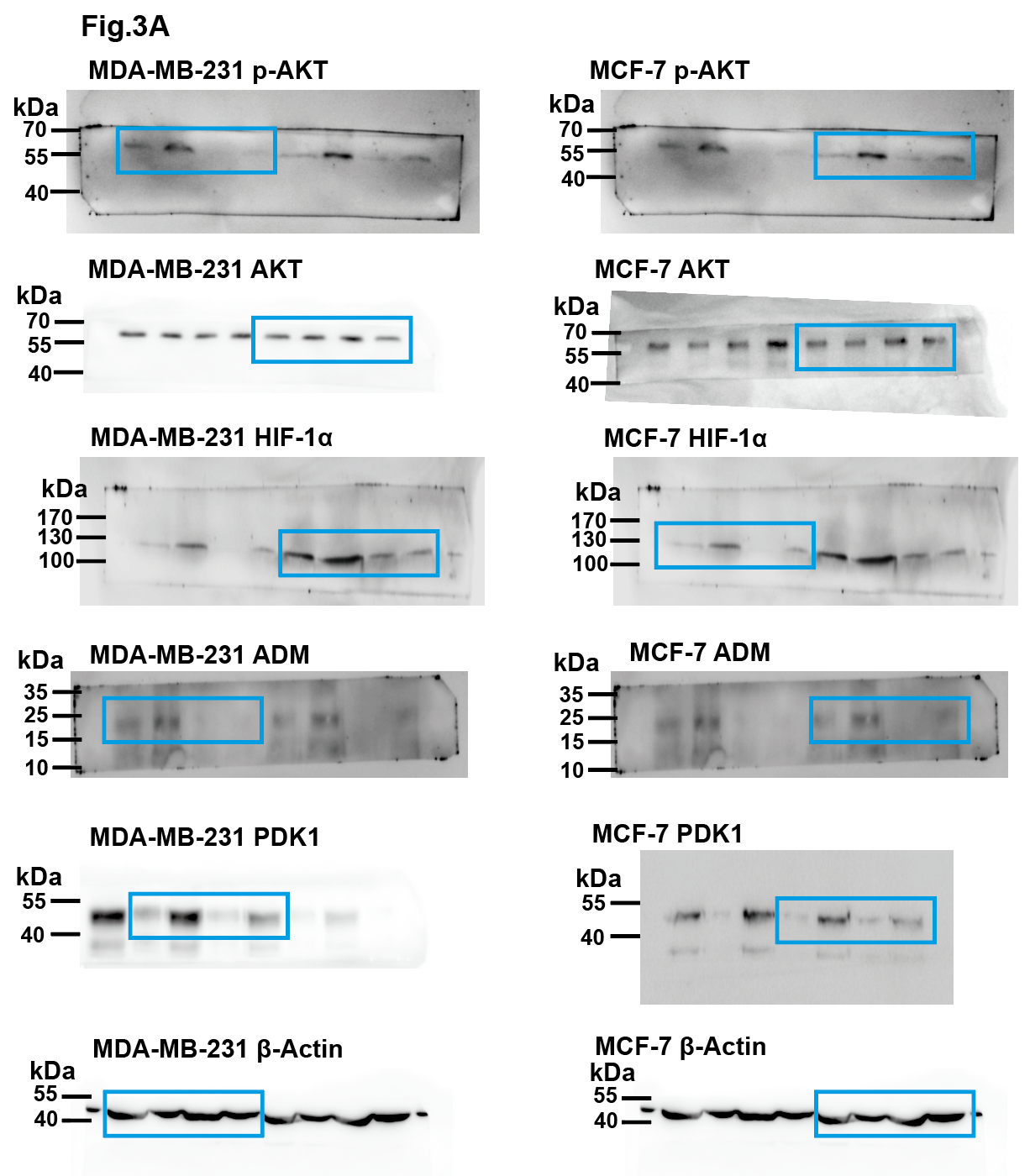


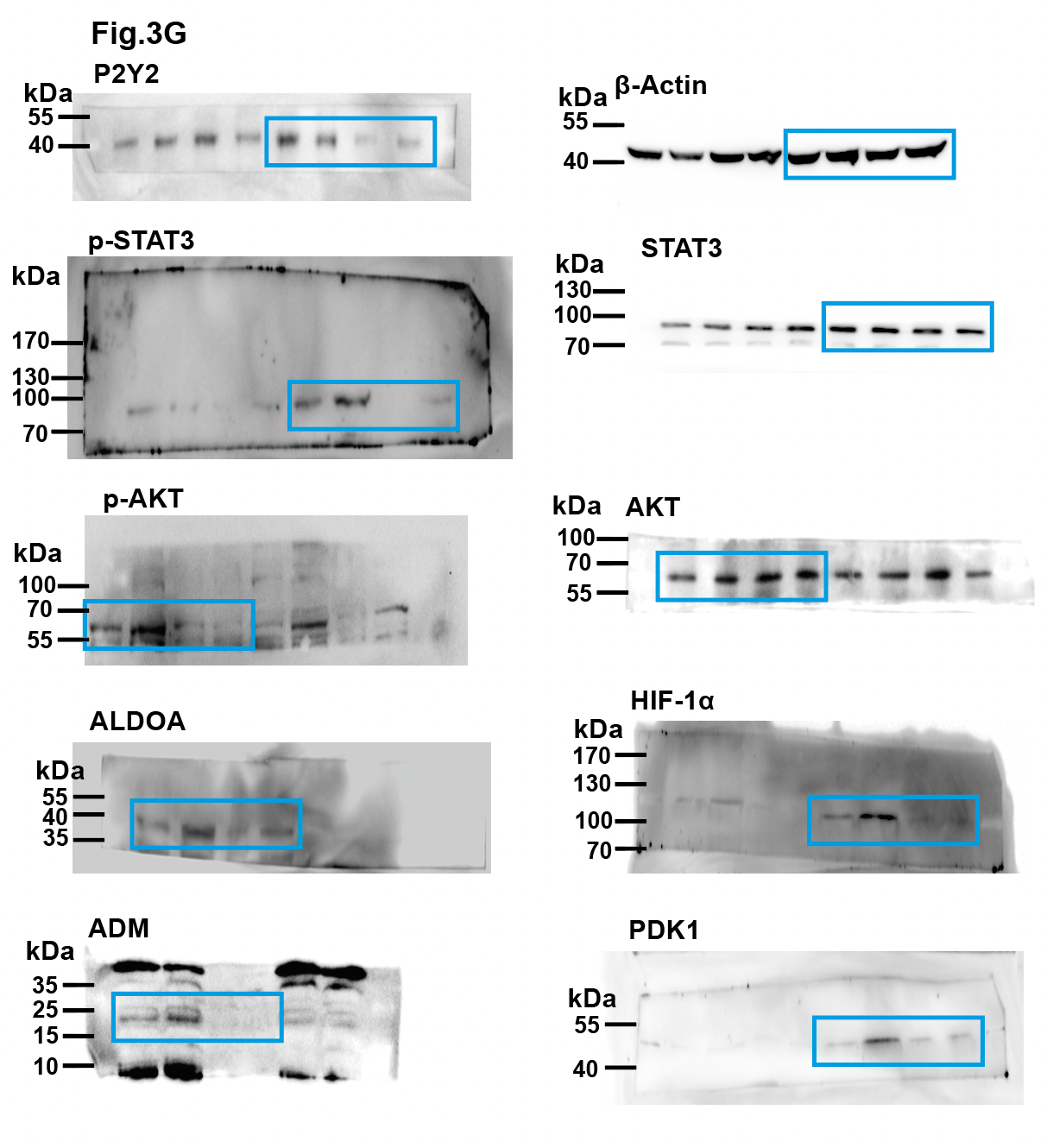


**Uncropped blots related to Figure 4**


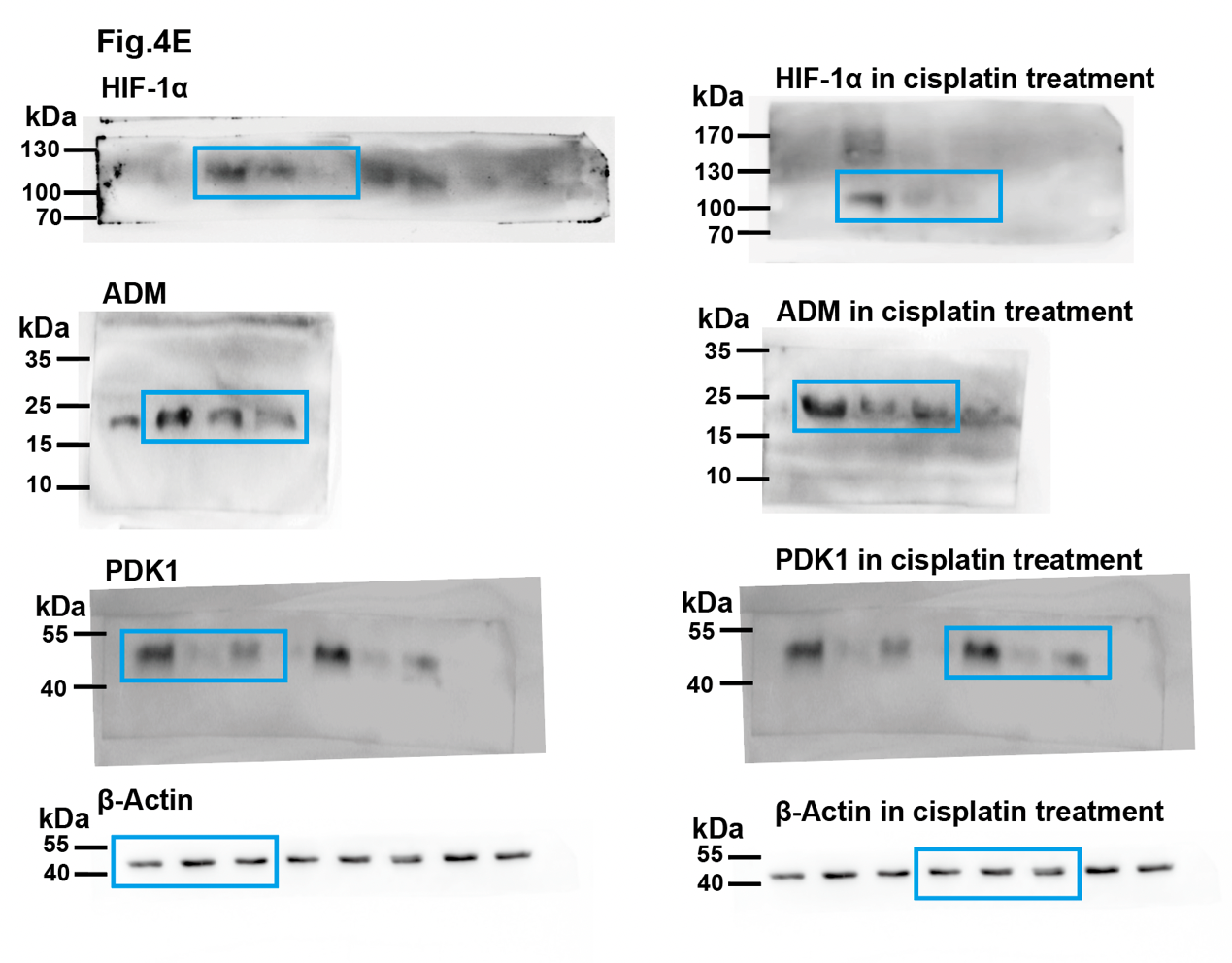


**Uncropped blots related to Figure 5**


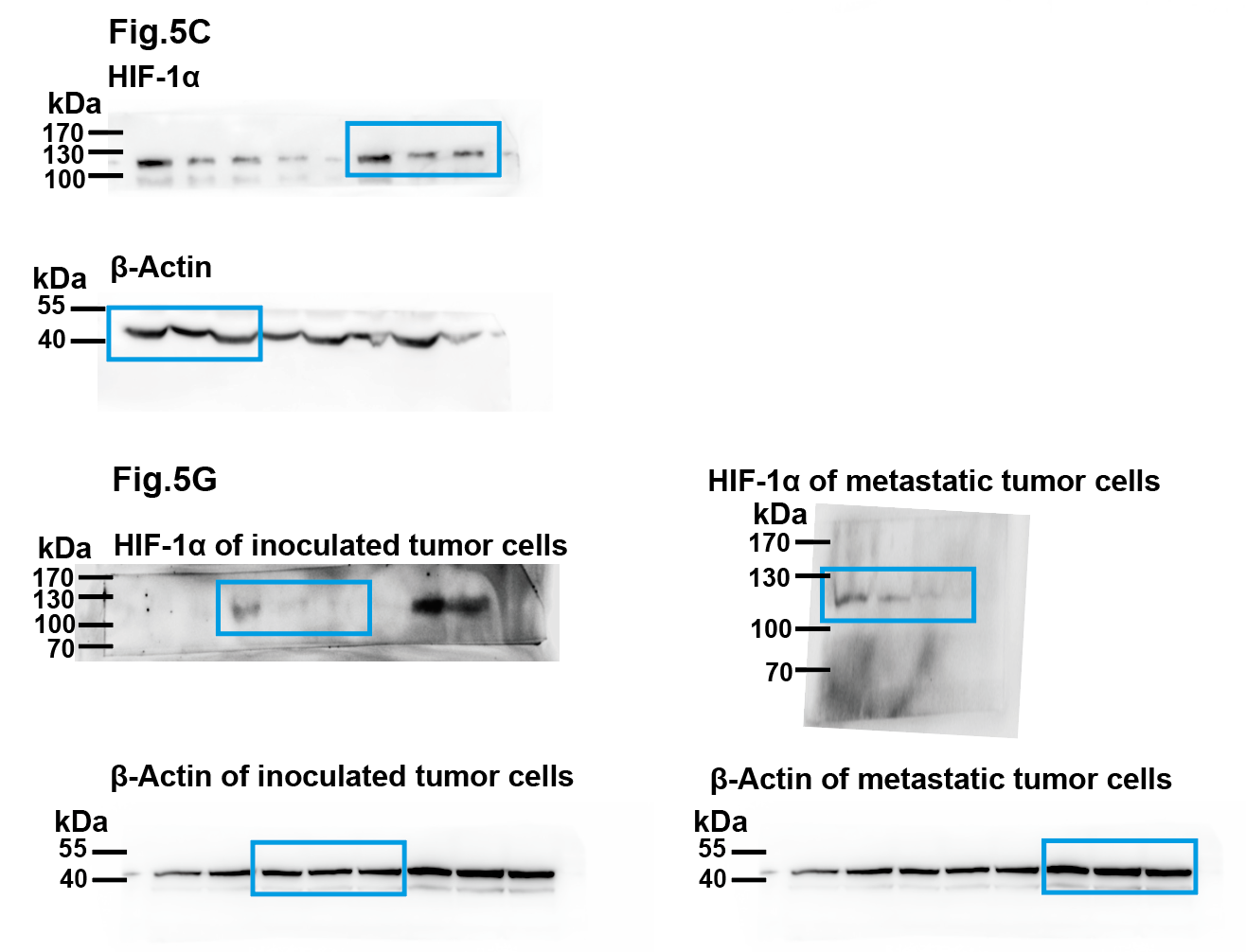


**Uncropped blots related to Figure 6**


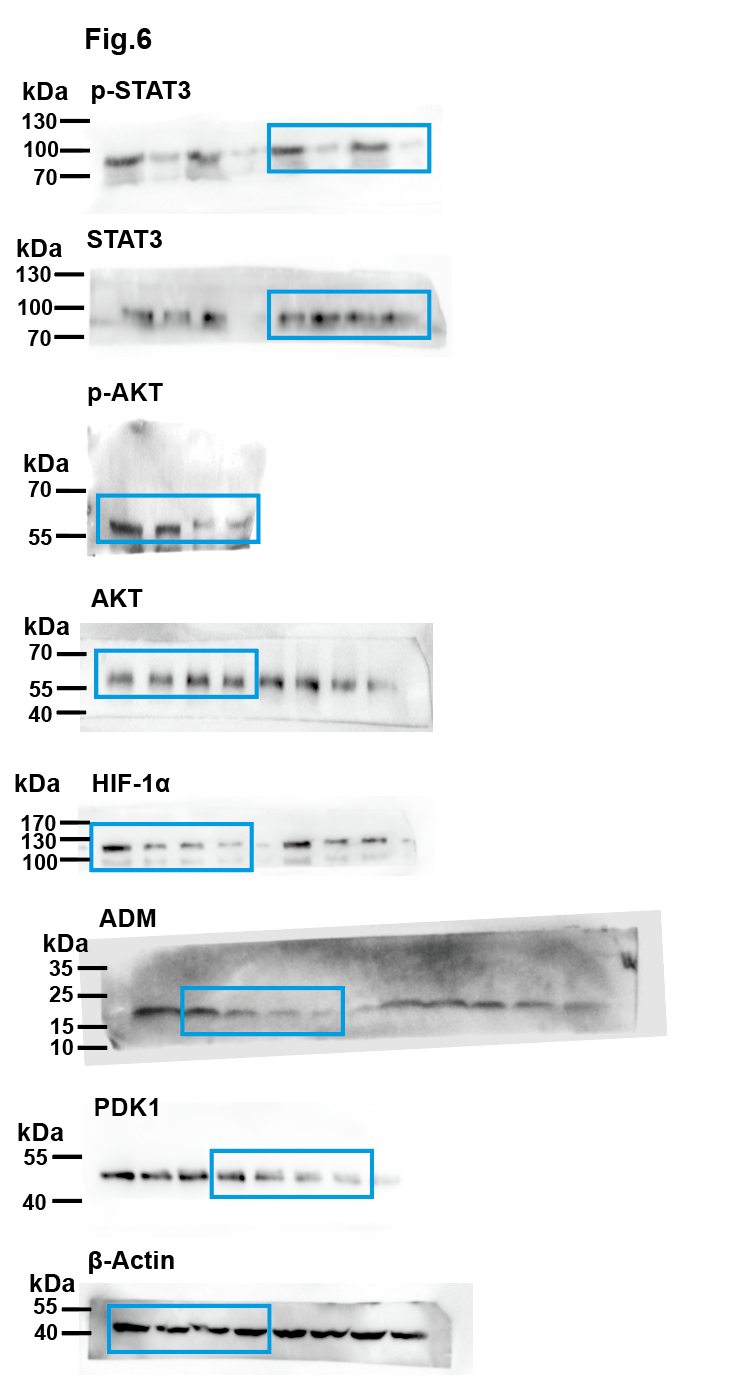


**Uncropped blots related to Figure S1**


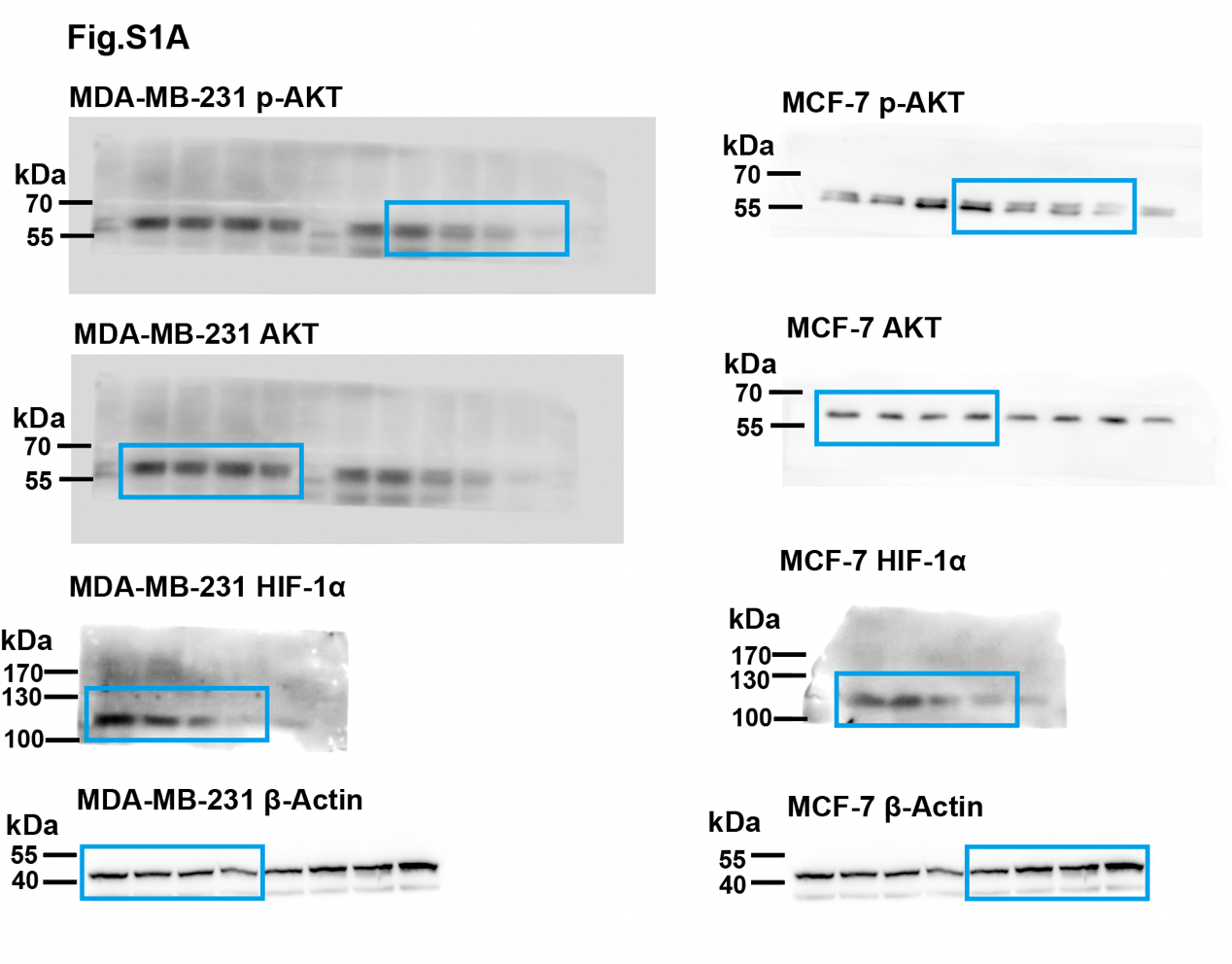


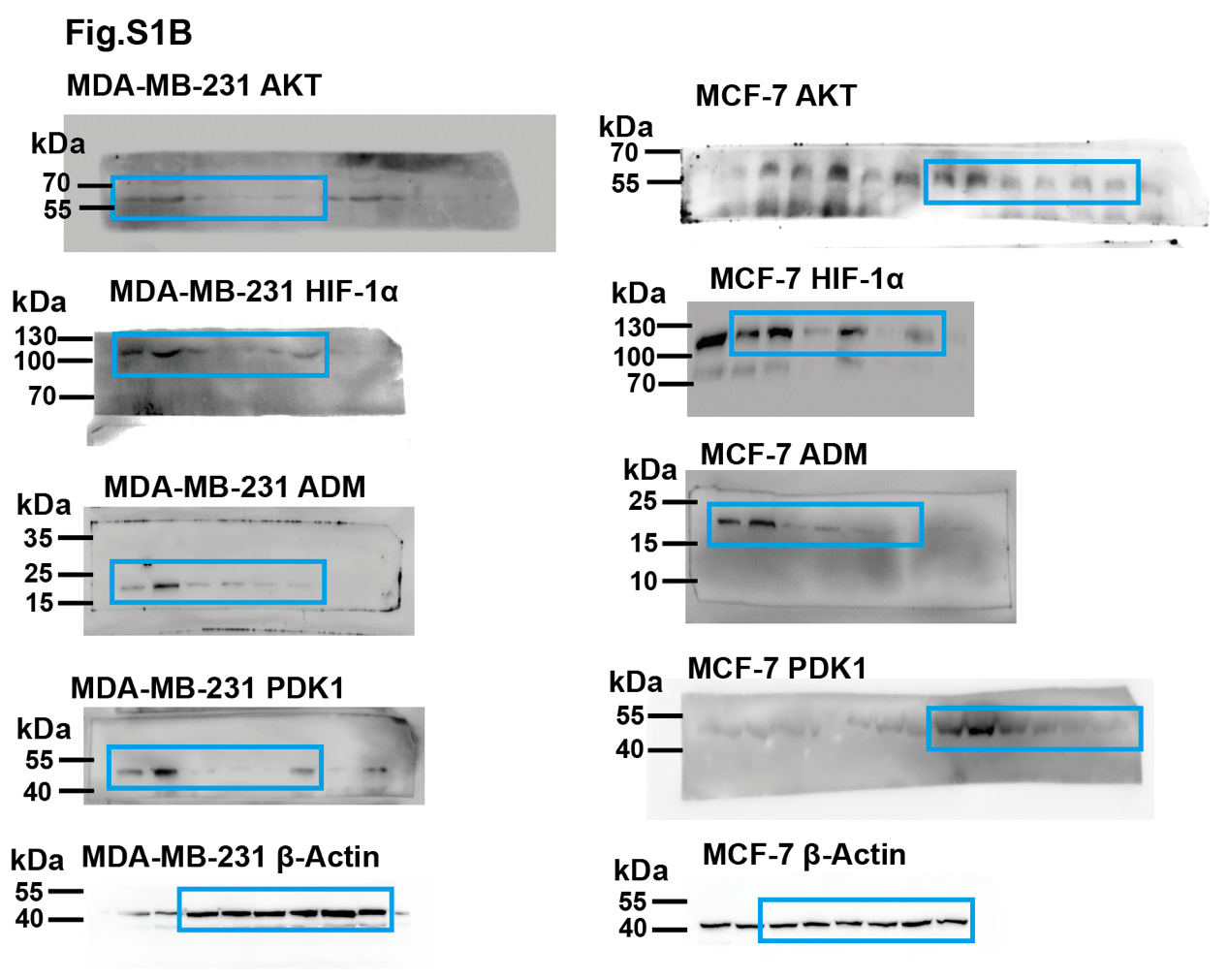


**Uncropped blots related to Figure S2**


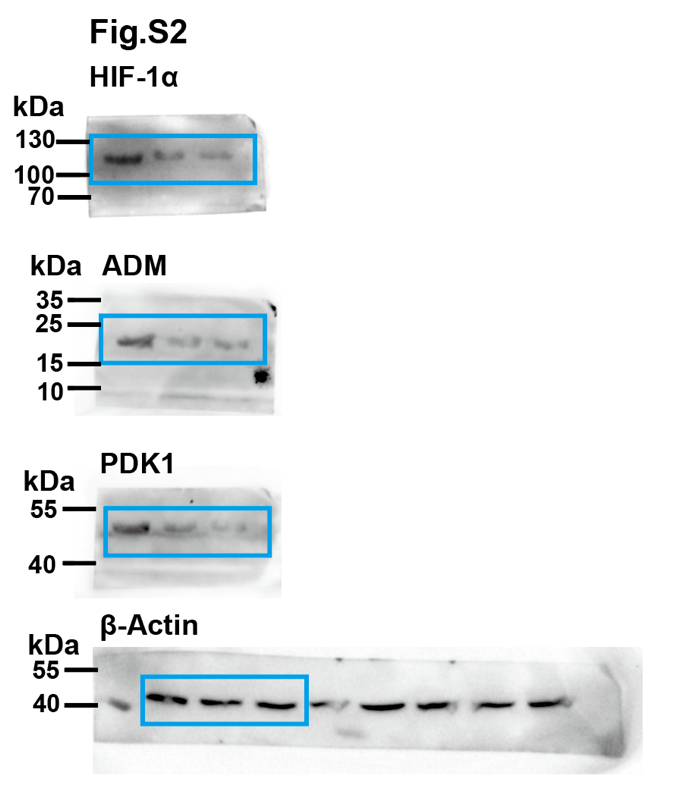


**Uncropped blots related to Figure S3**


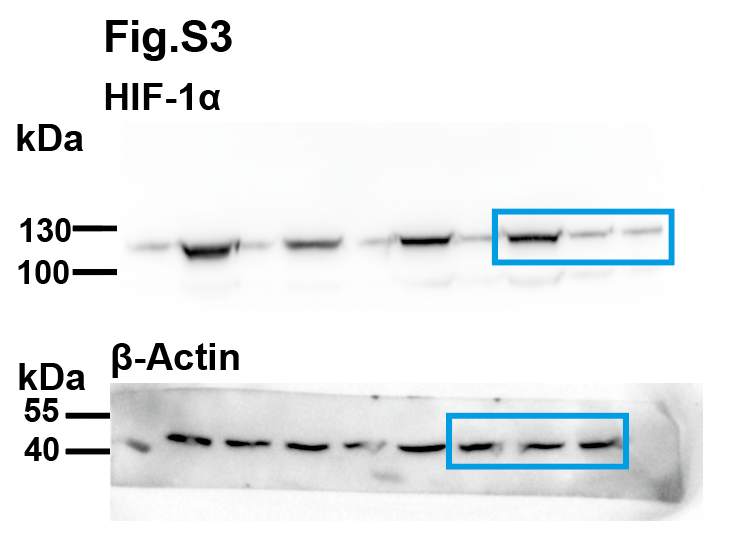


**Uncropped blots related to Figure S4**


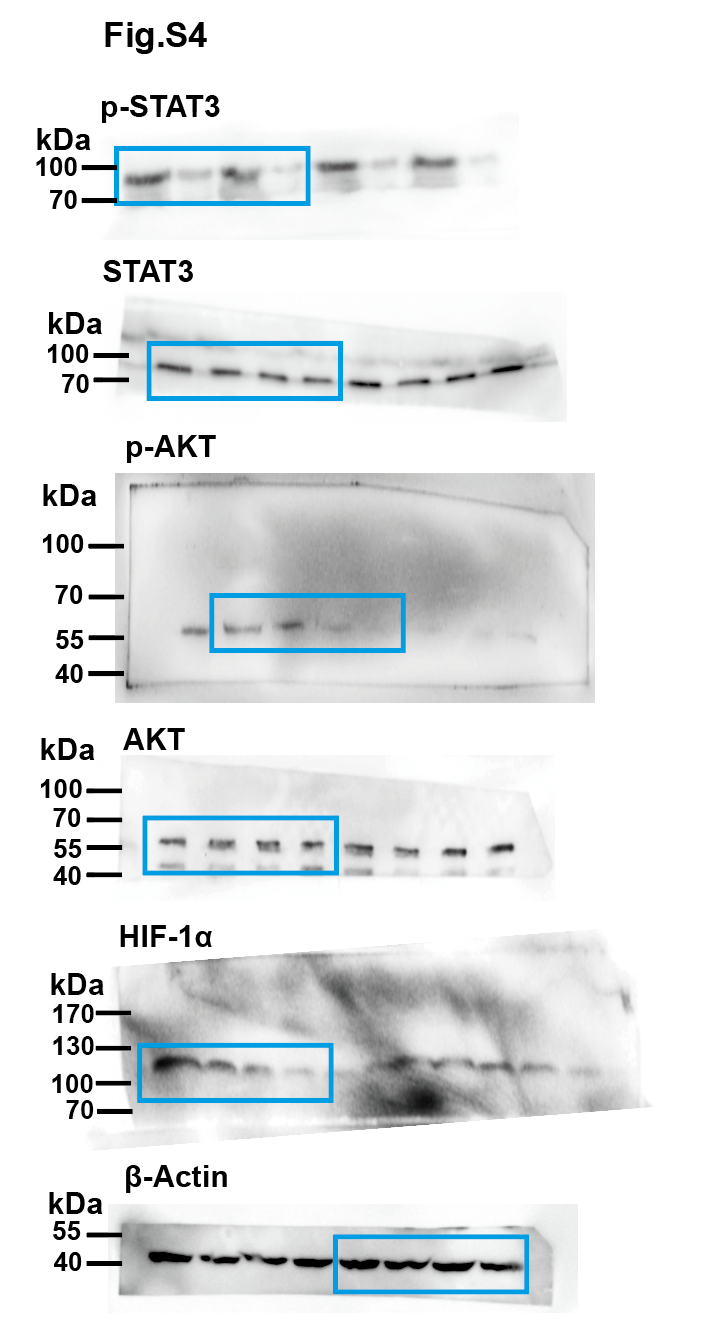

Supplement: Supplementary file 3 — Original Data File [file 41419_2022_4647_MOESM3_ESM.docx]
