# Supplementary material for: Extracellular ATP promotes breast cancer chemoresistance via HIF-1α signaling
Source: Cell Death Dis. 2022 Mar 2;13(3):199. doi: 10.1038/s41419-022-04647-6 (PMC8891368; doi:10.1038/s41419-022-04647-6)
Supplement: Supplementary file 4 — Supplementary Table 1 [file 41419_2022_4647_MOESM4_ESM.docx]

Supplementary Table 1: Mass spectrometry analysis of ALDOA at indicated band

| **Band** | **Identified proteins** | **Sequence** | **Area** | **q-Value** |
| --- | --- | --- | --- | --- |
| 39.4kDa | ALDOA | IGEHTPSALAIMENANVLAR | 8.289E8 | 0 |
|  |  | GILAADESTGSIAK | 1.673E9 | 0 |
|  |  | LQSIGTENTEENRR | 8.993E7 | 0 |
|  |  | ALANSLAcQGK | 9.674E8 | 0 |
|  |  | QLLLTADDR | 2.547E9 | 0 |
|  |  | cPLLKPWALTFSYGR | 3.093E8 | 0 |
|  |  | TVPPAVTGITFLSGGQSEEEASINLNAINK | 1.183E8 | 0 |
|  |  | ADDGRPFPQVIK | 8.570E8 | 0 |
|  |  | GVVPLAGTNGETTTQGLDGLSER | 2.537E8 | 0 |
|  |  | ELSDIAHR | 7.867E8 | 0.001 |
|  |  | YASIcQQNGIVPIVEPEILPDGDHDLKR | 1.328E8 | 0.002 |
|  |  | VLAAVYK | 1.698E9 | 0.004 |
|  |  | ALQASALK | 1.427E9 | 0.004 |
|  |  | AAQEEYVK | 3.551E8 | 0.004 |
